# JNK signaling regulates oviposition in the malaria vector *Anopheles gambiae*

**DOI:** 10.1038/s41598-020-71291-5

**Published:** 2020-09-01

**Authors:** Matthew J. Peirce, Sara N. Mitchell, Evdoxia G. Kakani, Paolo Scarpelli, Adam South, W. Robert Shaw, Kristine L. Werling, Paolo Gabrieli, Perrine Marcenac, Martina Bordoni, Vincenzo Talesa, Flaminia Catteruccia

**Affiliations:** 1 Dipartimento di Medicina Sperimentale, Università Degli Studi di Perugia, Sant’ Andrea Delle Fratte, Piano 4, Edificio D, Piazzale Gambuli 1, 06132 Perugia, Italy; 2 Department of Immunology and Infectious Diseases, Harvard T.H. Chan School of Public Health, 665 Huntington Avenue, Building 1, Room 103, Boston, MA 02115 USA; 3 Present Address: Verily Life Sciences, South San Francisco, CA 94080 USA; 4 Present Address: Dipartimento Bioscienze, University of Milan, 20133 Milan, Italy

**Keywords:** Hormones, Kinases, Malaria, Animal behaviour, Reproductive biology

## Abstract

The reproductive fitness of the *Anopheles gambiae* mosquito represents a promising target to prevent malaria transmission. The ecdysteroid hormone 20-hydroxyecdysone (20E), transferred from male to female during copulation, is key to *An. gambiae* reproductive success as it licenses females to oviposit eggs developed after blood feeding. Here we show that 20E-triggered oviposition in these mosquitoes is regulated by the stress- and immune-responsive c-Jun N-terminal kinase (JNK). The heads of mated females exhibit a transcriptional signature reminiscent of a JNK-dependent wounding response, while mating—or injection of virgins with exogenous 20E—selectively activates JNK in the same tissue. RNAi-mediated depletion of JNK pathway components inhibits oviposition in mated females, whereas JNK activation by silencing the JNK phosphatase *puckered* induces egg laying in virgins. Together, these data identify JNK as a potential conduit linking stress responses and reproductive success in the most important vector of malaria.

## Introduction

*Anopheles gambiae* mosquitoes are the most important vectors for *Plasmodium* malaria parasites, which infected at least 200 million people and caused more than 400,000 deaths in 2018^1^. The number of malaria deaths has more than halved since the year 2000 largely as a result of mosquito control strategies, especially insecticide-treated bed nets^1^. This promising progress is, however, threatened by the spread of insecticide resistance in *Anopheles* populations^2^, highlighting the pressing need for novel strategies for mosquito control. A number of recently proposed alternatives aim at reducing vector populations by regulating female reproductive output via either chemical^3^ or genetic^4^ means, and their successful development is dependent upon a detailed understanding of the mechanisms regulating reproduction in *Anopheles*^5^.

Mating represents a vulnerable step in the *An. gambiae* reproductive cycle as it happens only once in the female’s lifetime. During this single sexual event, males transfer sperm along with a gelatinous mating plug that contains a host of proteins and other factors produced by the male accessory glands^6,7^, including the ecdysteroid hormone 20-hydroxyecdysone (20E)^8,9^. Sexual transfer of this steroid hormone is a feature that is unique to anophelines, having evolved specifically in the *Cellia* subgenus and exclusively in the lineages leading to the most important African and South East Asian malaria vectors^10,11^. Transfer of 20E drives profound behavioral and physiological changes in the female collectively termed post-mating responses^9,12,13^. Perhaps the most striking of these changes are refractoriness to further mating, underpinning the female’s monandry, and a license to oviposit eggs developed following a blood meal^12^. Depletion of endogenous 20E levels in males reduces egg laying rates and increases remating frequency in the females with whom they mate^12^. Conversely, injection of exogenous 20E in virgin females is sufficient to induce both oviposition of developed eggs and refractoriness to further copulation^12^. Importantly, both refractoriness to further mating and the license to oviposit are irreversible, lifelong behavioral switches. However, the molecular processes through which 20E induces these changes in *An. gambiae* remain unknown.

Some insight into the mechanisms regulating these processes may come from the distantly related dipteran model organism, *Drosophila melanogaster*, where—similar to *An. gambiae*—the effects of mating include both a temporary refractoriness to further copulation and increased oviposition. Interestingly, while 20E in *D. melanogaster* does have an important role in regulating courtship behavior and specifically in the consolidation of long-term courtship memory in males^14,15^, post-mating responses in fruit fly females are not driven by 20E but by small male accessory gland peptides (Acps) transferred to the female during mating. These include ovulin, which is implicated in the control of oviposition^16^, and most importantly Acp70A, also known as Sex Peptide (SP)^17^. SP is necessary and sufficient to induce the post-mating switch: injection of exogenous SP induces both refractoriness to mating and oviposition^17^ while loss of the G protein-coupled Sex Peptide Receptor (SPR) in females largely blocks these responses^18^. Consistent with its role in modulating female post-mating behavior, SPR is found in neurons innervating the reproductive tract as well as the brain and ventral nerve chord^18,19^, while ovulin acts through octopamine-dependent neurons^16^. The importance of the brain in controlling female responses after copulation in *Drosophila* is also demonstrated by the fact that in genetic SPR mutants post-mating responses can be rescued by introducing a mutation that yields a leaky blood–brain barrier phenotype^20^, identifying the entry of mating factors into the brain as a potentially crucial step in inducing post-mating changes. Consistent with these findings, the *Drosophila* brain exhibits a robust transcriptional program following mating or injection of exogenous SP^21^.

Here we show that 20E-induced oviposition behavior in *An. gambiae* is partially regulated by c-Jun N-terminal kinase (JNK) signaling in the female head. We detect a strong, mating-induced transcriptional signature in female heads, enriched in immune genes and reminiscent of a JNK-dependent wound-healing response. Silencing multiple components of JNK signaling reduces oviposition rates of mated females, as well as those of virgin females injected with 20E. Conversely, JNK activation by depletion of the negative regulator *puckered* increases oviposition rates in virgin females. Our results unveil an unexpected link between an important mosquito reproductive behavior and the activation of JNK, a pathway classically associated with stress resistance and longevity^22,23^ but which is also pivotal to anti-plasmodium immunity^24,25^.

## Results

### A transcriptional signature of wounding response is found in the head after mating

To gain insight into the molecular basis of the mating response in *An. gambiae*, we performed transcriptional analysis of the heads of mated and age-matched virgin females at 3 and 24 h post mating (hpm). Microarray analysis identified a strong immune signature triggered by mating in the head, the like of which had not been detected in similar analyses of other *An. gambiae* female tissues^12,13,26^. As summarized in Table 1, 23 genes were differentially regulated after mating at the two time points under analysis, 22 of which were upregulated at either 3  hpm (11 genes) or 24  hpm (11 genes). A single gene, an acyltransferase (AGAP007078), was down regulated in the head 24  hpm. Functionally, 6 of the 22 upregulated genes were common to a group of genes previously linked to the wounding response in *An. gambiae*^27^, while 16 were implicated in the melanization pathway, which has been studied in this species predominantly in the context of *Plasmodium* infection^28,29^ but is also strongly linked to wound healing in other insects including *Drosophila*^30,31^. In addition, 13 of the upregulated genes were previously found to be preferentially expressed in hemocytes^32^, cells related to mammalian macrophages and central to the repair and regeneration of damaged cells^33^ and to the wounding-induced transcriptional response in *Drosophila*^34^.

**Table 1:** Genes regulated in the head after mating are enriched in genes linked to wound-healing, hemocytes and the JNK pathway.

Gene name	AGAP	Mating regulation	Link to melanization^28,29^	Upregulated by wounding^27^	Hemocyte-enriched^32^
**Upregulated genes**					
Melanization					
PPO2	AGAP006258	2.5-fold, 24 hpm *p* = 0.0082	✓		✓
PPO5	AGAP012616	1.8-fold, 24 hpm *p* = 0.0065	✓		✓
PPO6	AGAP004977	2.1-fold, 24 hpm *p* = 0.0070	✓		✓
CLIPB1	AGAP003251	1.7-fold, 3 hpm *p* = 0.012	✓		✓
CLIPB15	AGAP009844	2.8-fold, 24 hpm *p* = 0.011	✓	✓	✓
CLIPC7	AGAP003689	2.1-fold, 24 hpm *p* = 0.00049	✓	✓	✓
CLIPE11-like	AGAP003691	3.8-fold, 3 hpm *p* = 0.000018	✓	✓	✓
Gastrulation-defective	AGAP013252	1.2-fold, 24 hpm *p* = 0.0035	✓		
Serpin 17	AGAP001376	1.8-fold, 3 hpm *p* = 0.0016	✓		✓
Yellow F2	AGAP004324	2.4-fold, 3 hpm *p* = 0.0075	✓		✓
TEP/LRIM family					
TEP1	AGAP010815	3.1-fold, 24 hpm *p* = 0.0034	✓	✓	
TEP4	AGAP010812	1.7-fold, 24 hpm *p* = 0.00049	✓		✓
TEP8	AGAP010831	3.3-fold, 24 hpm *p* = 0.0012	✓		
TEP19	AGAP010832	3.3-fold, 24 hpm *p* = 0.0035	✓		
APL1C	AGAP007033	2.1-fold, 24 hpm *p* = 0.018	✓	✓	✓
Coagulation					
TGase2	AGAP009098	2.4-fold, 3 hpm *p* = 0.0095	✓	✓	✓
Other					
membrane protease	AGAP001365	1.5-fold, 3 hpm *p* = 0.0078			✓
Unknown	AGAP004316	3.6-fold, 3 hpm *p* = 0.0075			
Vesicle transport	AGAP006609	1.1-fold, 3 hpm *p* = 0.0084			
Carboxylsterase	AGAP011509	2.2-fold, 3 hpm *p* = 0.0029			
Hsc70	AGAP004192	2.5-fold, 3 hpm *p* = 0.017			
Hsp90b	AGAP001424	1.1-fold, 3 hpm *p* = 0.012			
**Down regulated genes**					
Acyltransferase	AGAP007078	1.5-fold, 3 hpm *p* = 0.012			

The 23 genes identified by microarray as being significantly regulated in the head after mating were compared with the relevant literature reports indicated. The mean mating-induced fold-change over age-matched virgins, the time point at which that change was observed and the adjusted *p* value, after False Discovery Rate (FDR) correction for multiple testing, are indicated (further detail of statistical validation of microarray data is included in “Methods”).

At the earlier 3  hpm time point we found mating-induced upregulation of transglutaminase 2 (TGase2), a member of an enzyme family involved in chemical crosslinking of proteins in the hemocel that is implicated in the coagulation responses that follow infection or trauma^31^ and was previously shown to be involved in JNK-dependent wounding responses in *An. gambiae*^27^ (Table 1). Also upregulated at 3  hpm were two members of the CLIP protease family, CLIPs B1 and 11E-like, serine proteases which initiate proteolytic cascades leading to the activation of prophenol oxidases (PPOs) that mediate both melanization^35^ and coagulation^31^. We also identified the CLIP inhibitor, Serpin 17, a member of the serine protease inhibitor (Serpin) family which block CLIP-mediated proteolytic processing of PPO enzymes^36^. Finally, we detected the up-regulation of L-dopachrome tautomerase (also known as Yellow F2), an enzyme involved in melanin biosynthesis^35^ (Table 1).

Among the genes upregulated at 24  hpm we identified three PPOs (PPO2, PPO5, and PPO6) and three additional CLIP proteases; CLIP15B, CLIPC7, and *gastrulation defective*. Beyond the melanization pathway we also found multiple thioester-containing proteins (TEP1, TEP4, TEP8 and TEP19) which are linked to the mosquito complement-like response^37^, along with APL1C (*Anopheles-Plasmodium*-responsive leucine-rich repeat protein 1, isoform C), a member of the leucine-rich repeat (LRR) immune protein (LRIM) family. APL1C associates with another LRIM family member (LRIM1, not identified here), and TEP1 in a complex that retains TEP1 in a stably active form that can then form thioester bonds with surface-exposed proteins of invading pathogens including *Plasmodium* parasites^38–40^, targeting them for lysis. Taken together, these data suggest mating induces a strong wounding response in the head of *An. gambiae* females.

### JNK is activated in the head after mating

**Figure 1:**
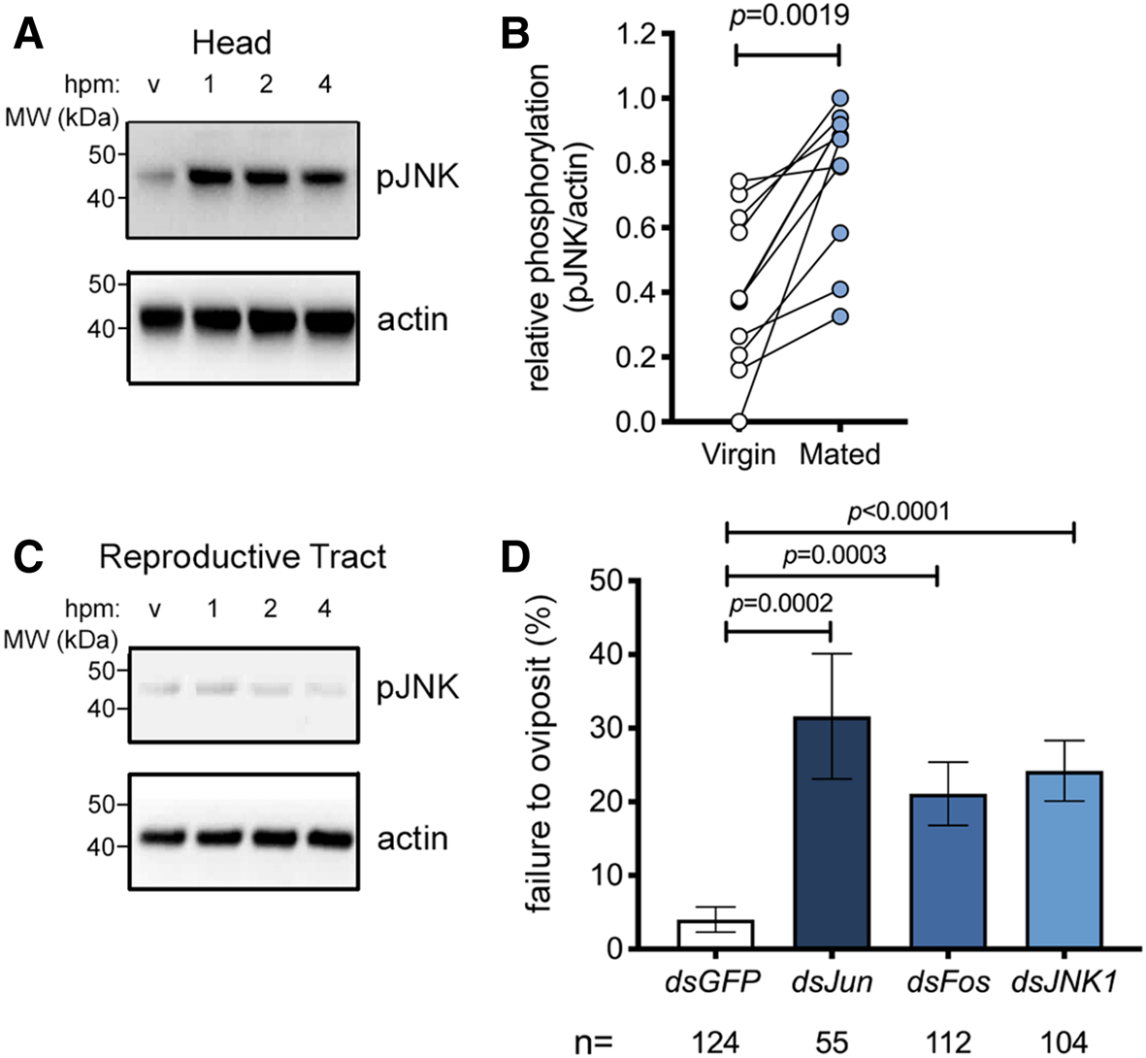
JNK is activated in the head after mating and required for mating-induced oviposition. **A**–**C **Representative Western blot of extracts of (**A**) heads or (**C**) reproductive tracts (ovaries, atrium and spermatheca) prepared from 3-day-old virgin or mated (plug-positive) females (10–15 tissues/point) at 1, 2, and 4 h post mating (hpm). Tissue extracts were subjected to Western blot analysis with anti-pJNK then stripped and re-probed with anti-actin as loading control. In (**B**), the optical density of bands was quantified (ImageJ) in eleven similar experiments, and the pJNK signal was normalized against actin and expressed as ‘relative phosphorylation’ in virgins (white circles) and females 2  hpm (blue circles). Connected circles represent data from a single experiment. Differences between treatment groups were analyzed using a Mann–Whitney test and significant *p* values (*p* < 0.05) reported. (**D**) RNAi silencing of JNK (ds*JNK*), Jun (ds*Jun*) or Fos (ds*Fos*) prior to mating reduces the oviposition rates of mated (plug-positive) females. The graph shows the percentage of females (mean ± SEM from 9 independent biological replicates, each comprising 16–83 females) failing to oviposit by day 4 post mating, analyzed using a logistic regression test. For the dataset as a whole, chi-squared = 49.1, *p* < 0.0001.

The preponderance of wounding-related transcripts in the head after mating prompted us to ask what signaling pathways might lead to such a response. The importance of the JNK signaling pathway in the insect wounding response has been highlighted in both *An. gambiae*^27^ and *Drosophila*^41^. Since JNK is well known to be activated post-transcriptionally by phosphorylation, we asked whether mating might increase levels of active (phosphorylated) JNK (pJNK) in the female’s head. *An. gambiae* females were dissected around the onset of detectable transcriptional changes (1–6  hpm), and heads were analyzed by Western blot using an antibody specifically recognizing pJNK. Mating induced a marked increase in the levels of pJNK in the head compared to virgin controls, an effect that was already detectable at 1  hpm and was still evident at 4  hpm across multiple experiments (Fig. 1A,B). In mated females we detected a strong band at 46 kDa and a much weaker band at 52 kDa, similar to reports in *An. stephen*si^42^. This mating-induced JNK response was tissue-specific as we did not detect it in other tissues including the reproductive tract (ovaries, atrium and spermatheca) or the rest of the body (Fig. 1C, Supplementary Fig. S1). Moreover, while levels of pJNK increased in the heads of mated females (one sample t test, p = 0.0496, n = 4), levels of the phosphorylated (active) form of extra-cellular signal-regulated kinase (pERK), a second member of the MAP kinase family to which JNK belongs, albeit one traditionally associated with growth stimuli^43^, were reduced in mated heads relative to virgin controls (one sample t test, *p* = 0.02, n = 4) (Supplementary Fig. S1), suggesting a level of pathway specificity.

### JNK1 is required for mating-induced oviposition

To determine the contribution of the JNK pathway to post-mating responses, we silenced elements of the pathway by RNAi and examined the effect on oviposition, a physiological response that is induced in mated females once eggs are fully developed after blood feeding. Given the annotation of two distinct *An. gambiae JNK* genes, *JNK1* (AGAP029555) and *JNK3* (AGAP009460), we performed preliminary experiments to assess the relative expression of each transcript and found that *JNK1* transcript levels were 2–3 orders of magnitude more abundant than *JNK3* in all tissues measured (Supplementary Fig. S2) leading us to focus on the *JNK1* gene product. Thus, virgin females were injected with ds*RNA*s targeting *JNK1* (*dsJNK1*, Supplementary Fig. S3) or its two transcription factor targets, *Jun* (ds*Jun*) and *Fos* (ds*Fos*), using ds*GFP* as control. Using a previously established protocol^12^, injected females were then blood-fed and mated, and oviposition rates were measured. *JNK1* silencing inhibited the mating-induced increase pJNK in the head (Supplementary Fig. S4) and significantly reduced oviposition compared to control females (logistic regression model, *p* < 0.0001, Fig. 1D). The failure to oviposit after mating was 5.6-fold more likely in *dsJNK1*-treated females than in *dsGFP*-treated controls (odds ratio [OR] = 5.6; *p* < 0.0001). Similarly increased rates of oviposition failure were observed with *dsFos* (OR = 4.03, *p* = 0.0003) and ds*Jun* (OR = 6.2, *p* = 0.0002) (Fig. 1D). We observed no significant effect on the total number of eggs developed or the number of eggs oviposited (Supplementary Fig. S5) in any group, and similarly, gene silencing did not affect the ability of females from any of the groups to mate, as assessed by the detection of mating plugs in the atrium (*dsGFP,* 218 plug-positive females/ 274 females captured *in copula* [79.6%]; *dsJun* 83/108 [76.9%, *p* = 0.57, Fisher’s exact test); *dsFos* 207/262 [79.0%. *p* = 0.92]; *dsJNK*, 187/252 [74.2%, *p* = 0.15]). Finally, *JNK1* depletion reduced the up-regulation of wounding-related genes (*APL1C, TEP1* and *PPO2*) in the female head after mating (Supplementary Fig. S6). Together, these data suggest the involvement of the JNK pathway in the mating-induced cascades leading to oviposition in *An. gambiae*.

### Depletion of the JNK-phosphatase puckered is sufficient to induce oviposition in virgins

**Figure 2:**
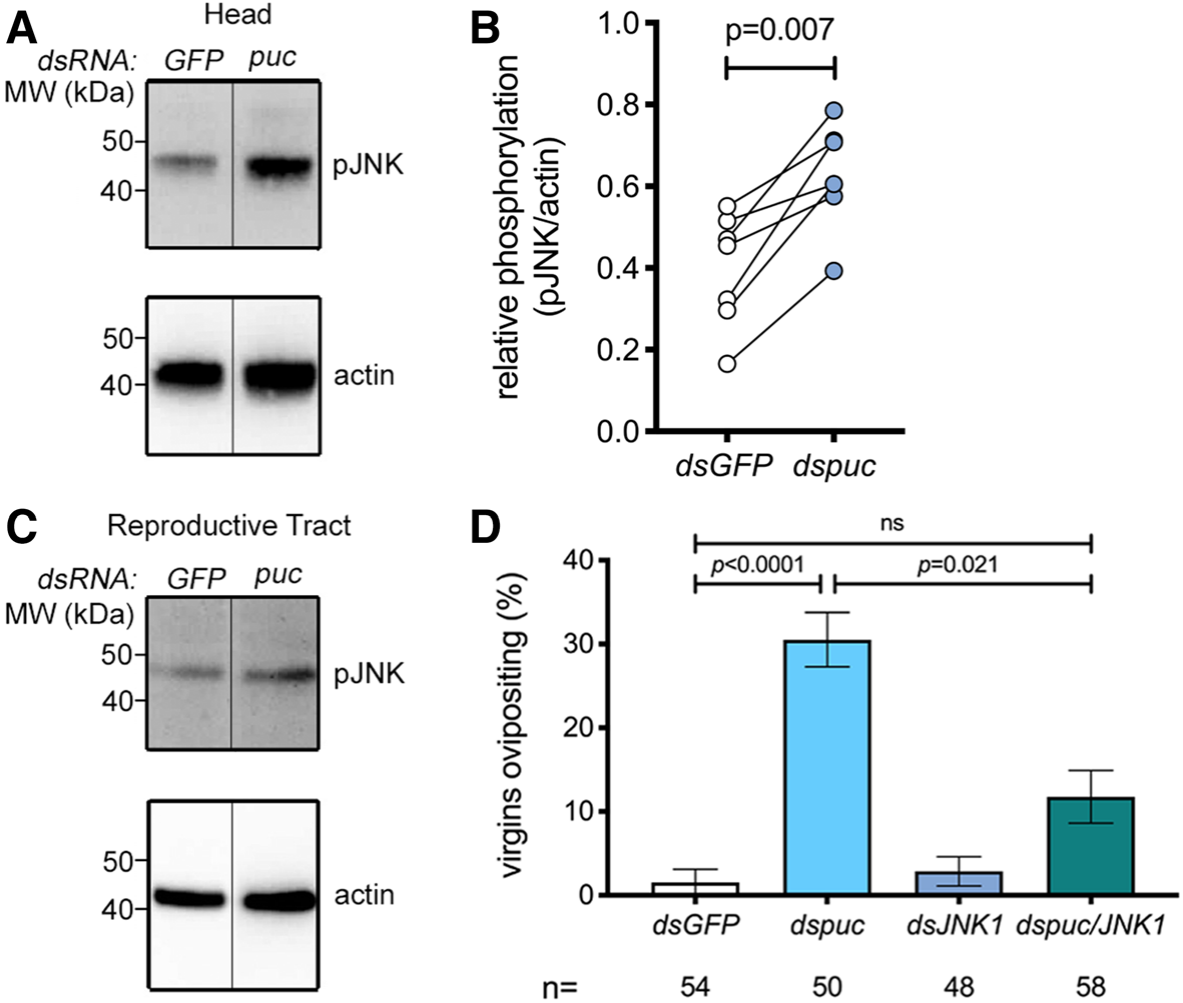
*Puckered* knock down induces phospho-JNK in the head and JNK1-dependent oviposition in blood-fed virgins. (**A**–**C**) Representative Western blot of extracts of (**A**) heads or (**C**) reproductive tracts (ovaries, atrium and spermatheca) dissected from 10 to 15 virgin females injected with either *dsGFP* (*GFP*) or *dspuc* (*puc*) 48 h post injection (hpi), using anti-pJNK then stripped and re-probed with anti-actin as loading control. The line between *GFP* and *puc* indicates the removal of an unrelated intervening lane. In (**B**), the optical density of bands was quantified (ImageJ) in seven similar experiments, and the pJNK signal was normalized against actin and expressed as ‘relative phosphorylation’ of pJNK in *dsGFP*- (white circles) and *dspuc*-treated females (blue circles). Connected circles represent data from a single experiment. Differences between treatment groups were analyzed using a Mann–Whitney test and significant *p* values (*p* < 0.05) reported. (**D**) RNAi silencing of *puc* (*dspuc*) induces oviposition in blood fed virgins. Virgin females were injected with *dsGFP, dsJNK, dspuc* or jointly injected with *dsJNK and dspuc*, blood-fed and then placed in oviposition cups. The graph shows the percentage of females (mean ± SEM from 4 independent biological replicates, each comprising 20–75 females) successfully ovipositing by day 5 post blood feeding, analyzed using a logistic regression test. For the dataset as a whole chi-squared = 28.4, *p* < 0.0001.

Having established a role for the JNK pathway in mating-induced oviposition, we went on to determine whether an increase in the level of pJNK in the head might be sufficient, per se, to induce oviposition in blood-fed virgin females. The activation of JNK is regulated by the dual phosphorylation of its TxY motif by the MAP kinase kinase (MAP2K) Hemipterous, and is prevented by dephosphorylation of the same motif by the dual specificity phosphatase Puckered (Puc), also known as MAP kinase phosphatase 5 (MKP5). Because depletion of *puc* in *Drosophila* leads to the spontaneous activation of JNK-responsive genes^44^, we reasoned that silencing of this phosphatase might mimic the activation of JNK noted after mating. Indeed, *puc* silencing (Supplementary Fig. S3) increased pJNK levels relative to controls in the female head across multiple experiments (Fig. 2A,B), while no noticeable effects were observed in the reproductive tract of the same females suggesting tissue-specific activation (Fig. 2C). Moreover, a significant proportion of virgin females (logistic regression model, *p* < 0.0001) injected with *dspuc* laid eggs after blood feeding (Fig. 2D). Using the logistic regression model applied above, we determined that ds*puc* virgins were approximately 25-fold more likely to oviposit than controls (OR 24.6, *p* < 0.0028). These results are consistent with our findings that JNK activation in the head after mating induces oviposition.

While in *Drosophila* Puc acts selectively to regulate the JNK pathway^44^, a recent study in *An. stephensi* suggested that the same phosphatase can impact MAP kinases other than JNK^42^. To address the role of JNK1 in the oviposition induced by *dspuc*, we performed a double knock down of *puc* and *JNK1*. Consistent with a predominant role for JNK signaling in regulating this behavior, coinjection of *dspuc* with *dsJNK1* significantly reduced the frequency of oviposition induced by *dspuc* alone (OR relative to *dspuc* alone 3.7, *p* = 0.021) without affecting the efficiency of *puc* knock down (Supplementary Fig. S3). These data suggest that activation of JNK in the head of a virgin female is sufficient to induce oviposition.

### The JNK pathway is required for oviposition induced by exogenous 20E

**Figure 3:**
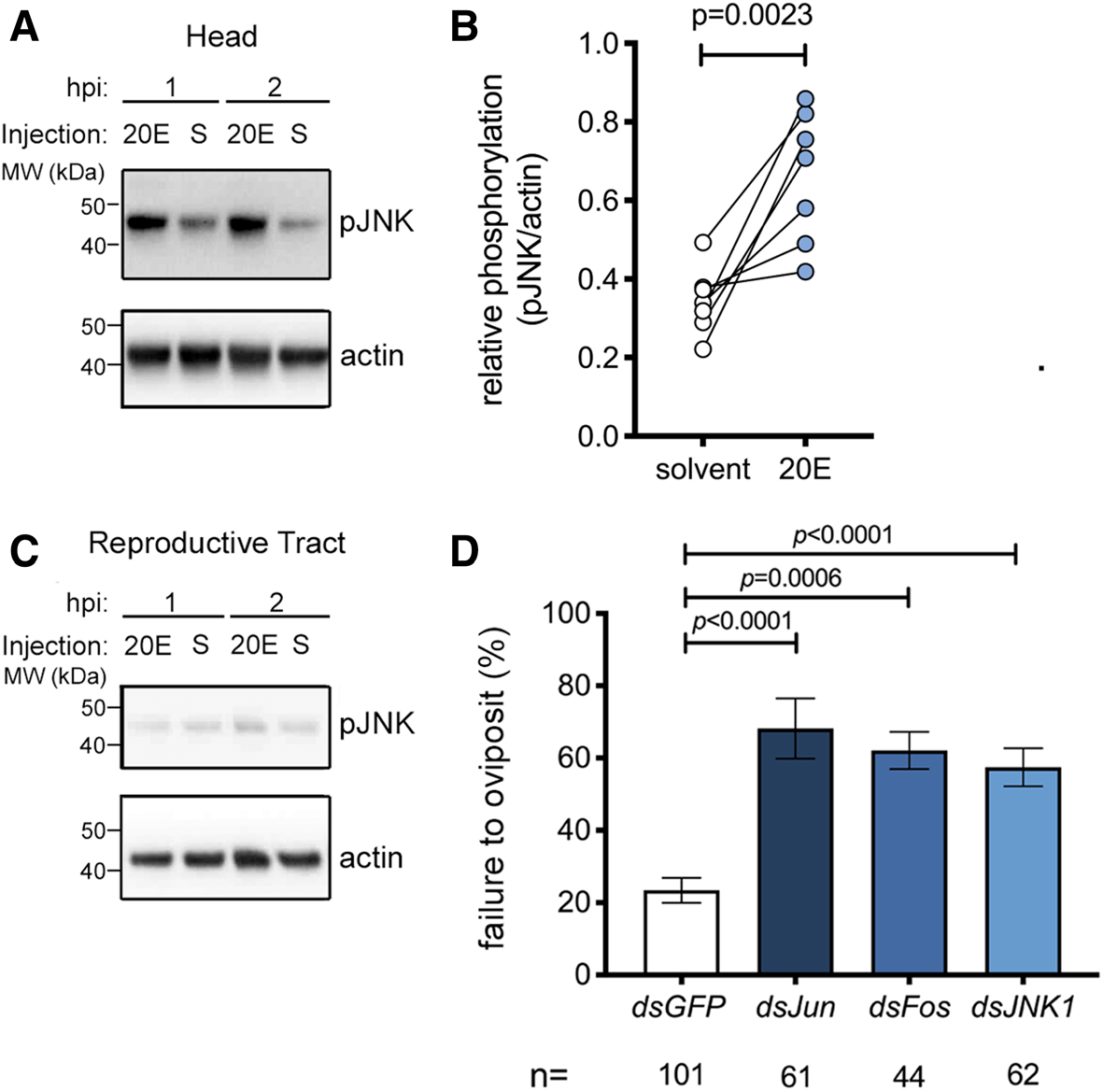
JNK pathway depletion causes failure of 20E-induced oviposition in blood-fed virgins. (**A**–**C**) Representative Western blot of extracts of (**A**) heads or (**C**) reproductive tracts (ovaries, atrium and spermatheca) dissected from 3-day-old virgin females (10–15 pooled tissues/point) injected with either 20E or a solvent control (S) at 1 or 2 h post injection (hpi) using anti-pJNK, and anti-actin as loading control. In (**B**), the optical density of bands was quantified (ImageJ) in seven similar experiments, and the pJNK signal was normalized against actin and expressed as ‘relative phosphorylation’ of pJNK in females injected with solvent (white circles) or 20E (2 hpi, blue circles,). Connected circles represent data from a single experiment. Differences between treatment groups were analyzed using a Mann–Whitney test and significant *p* values (*p* < 0.05) reported. (**D**) Virgin females were injected with ds*JNK*, ds*Jun* or ds*Fos*, blood-fed and then injected with 20E or solvent control and placed in oviposition cups. The graph shows the percentage of females (mean ± SEM from 7 independent biological replicates, each comprising 15–65 females) failing to oviposit by 4 days post 20E injection, analyzed using a logistic regression test. For the dataset as a whole chi-squared = 43.5, *p* < 0.0001.

Since thoracic delivery of exogenous 20E is sufficient to induce oviposition in blood-fed virgin females^12^, we next assessed whether injected 20E—like mating (Fig. 1A,B)—might also induce an increase in pJNK levels in the head. When compared to controls, a robust increase in pJNK in the head was observed across multiple experiments 1–2 h post injection (hpi) (Fig. 3A,B), while the reproductive tract again showed no JNK activation at these time points (Fig. 3C).

To determine if 20E-induced oviposition occurs via JNK signaling, we silenced *JNK1*, *Jun* or *Fos* and allowed females to take a blood meal. After completion of egg development, females were injected with either 20E or a solvent control, and oviposition rates were measured. The number of females failing to oviposit after 20E-injection increased markedly after treatment with *dsJNK1* (logistic regression model, *p* < 0.0001), compared to *dsGFP*-treated control females (OR of failed oviposition compared to *dsGFP* control = 5.4, *p* < 0.0001). Similar results were obtained after silencing of *Jun* (OR 5.8, *p* < 0.0001) and *Fos* (OR 4.1, *p* = 0.0006) (Fig. 3D). These data support the involvement of the JNK pathway in oviposition induced by sexual transfer of the male steroid hormone 20E. Injection of a solvent control failed to induce oviposition in any ds*RNA* group (94–100% failed oviposition, see Supplementary Table S1), as expected^12^.

## Discussion

The transfer of the ecdysteroid hormone 20E from *An. gambiae* males to females during copulation is linked to a life-long license to oviposit eggs developed across multiple gonotrophic cycles^10,12^. Here, we identify the JNK pathway, traditionally associated with responses to diverse environmental stressors including wounding^27^ and *Plasmodium* infection^24,25^, as a key component of the downstream events linking sexual transfer of 20E to oviposition. We document a rapid increase in the amount of active pJNK in the heads of mated females that is recapitulated by injection of exogenous 20E. The activation of JNK in the head after mating appears pathway-specific since ERK, an independently-regulated, growth-associated member of the MAP kinase family to which JNK belongs, is not activated in the same tissue. Moreover, JNK itself is not activated by mating in other tissues studied (reproductive tract or rest of body). The lack of a detectable JNK response to mating in the reproductive tract tallies with the absence of an obvious immune- or wounding-related gene signature in our previous microarray studies of mating-regulated genes in that tissue^12,13,26^, in contrast to the striking immune signature and copulatory wounding reported in *Drosophila* reproductive tract after mating^45,46^.

We find that the activation of the JNK pathway in the head is both necessary and sufficient for at least one element of the post-mating response—the oviposition of developed eggs. RNAi-mediated depletion of *JNK1* inhibited the mating-induced pJNK signal (Supplementary Fig. S4) and reduced oviposition induced either by mating (Fig. 1D) or by the injection of exogenous 20E (Fig. 3D). The link between this reduced egg laying phenotype and JNK function is highlighted by the fact it was phenocopied by depletion of *Jun* or *Fos*, transcription factor targets of pJNK. Moreover, activation of the JNK pathway—by RNAi-induced depletion of *puc—*was sufficient to both increase pJNK levels in the head (Fig. 2A,B) and trigger oviposition in blood-fed-virgin females (Fig. 2D). Importantly, while recent data have shown that in some settings Puc is capable of modulating MAP kinase pathways other than JNK^42^, our data show that the induction of oviposition by depletion of *puc* is largely reversed by concomitant depletion of JNK1, identifying this MAP kinase as the dominant Puc target in regulating this phenotype (Fig. 2D).

Despite the 100–1,000-fold greater transcript abundance of *JNK1 *(Supplementary Fig. S2), we cannot exclude a contribution to the oviposition phenotype from the *JNK3* gene product*,* given the 70% identity between the two over the mRNA sequence targeted by *dsJNK1*. Similarly, the origin and importance of the two protein bands observed using the pJNK antibody remain unclear. They could reflect the products of the *JNK1* and *JNK3* genes as suggested by others^42^ but might equally represent alternative post-translationally modified forms of the same gene product. Future studies using specific antibodies or gene targeting strategies will help address these questions.

Given the partial nature of the effects observed, our data also highlight the likely existence of JNK-independent pathways controlling oviposition, although we cannot exclude that these effects are due to incomplete gene silencing. Importantly, in the absence of tissue-specific gene knockout studies, the systemic nature of RNAi makes it difficult to exclude the possibility that JNK signaling outside the head may contribute to the regulation of oviposition, notwithstanding the head-specific activation of JNK observed after mating, 20E injection or *puc* depletion discussed above. The effects of the JNK pathway appear to affect oviposition rather than oogenesis, since neither the number of eggs developed or laid are affected by JNK pathway depletion ( Supplementary Fig. S5). Also, we observed no effects on the mating ability of JNK-depleted females, although it remains to be determined whether this pathway may affect female remating frequencies.

While, to our knowledge, this is the first demonstration that the stress-responsive JNK pathway is involved in an important reproductive behavior such as oviposition, egg laying has been previously linked to stress responses in mosquitoes. Stressful stimuli including heat, dessication, starvation and infection have all been shown to impact on the timing of oviposition^47–49^, whereas confinement stress has been hypothesized to increase the frequency of oviposition in various anophelines^50^. Links between steroid hormones and stress responses have also been documented^51–53^. Of particular relevance, 20E titers have been shown to increase following stressful social interactions such as courtship^15^. This has led to the proposal that—akin to related sex steroids in mammals^54^—20E functions in insects to consolidate stress-associated memory and to drive pathways of neuronal remodeling underpinning the development of appropriate adaptive behaviors^14^.

Although the mating-mediated mechanisms controlling egg laying remain largely elusive, the data presented here provide clear evidence of collaboration between 20E and the JNK pathway to induce oviposition. In fact, such collaboration is well documented during larval/pupal metamorphosis in *Drosophila*, where 20E and the JNK pathway together drive waves of apoptosis which control the reshaping or destruction of obsolete larval tissues^55^ as well as the remodeling of neuronal connections in the larval brain by axon and dendrite pruning^56^. It is plausible that an irreversible process of this type might regulate the lifelong behavioral changes induced by mating in *An. gambiae.* In future studies, it will be important to examine whether other elements of the post mating response, such as mating refractoriness, show a similar requirement for the JNK pathway. In addition, given the importance of the JNK pathway in the *An. gambiae* immune response to some *Plasmodium* infections^24,25^ and the centrality of 20E to mosquito physiology and parasite development^57^, it will be informative to examine whether 20E signaling in other contexts (e.g. after blood feeding) also engages with the JNK pathway.

## Methods

### Rearing of *Anopheles gambiae* mosquitoes

*An. gambiae* mosquitoes of the G3 line were maintained at 28 °C, 70% relative humidity with a 12 h light/ dark diurnal cycle and water and 10% glucose solution ad libitum and fed weekly on human blood obtained from the blood bank of the Servizio Immunotrasfusionale, Ospedale Santa Maria della Misericordia di Perugia. For mating experiments, males and females were maintained as virgins by separation of male and female pupae by microscopic examination of the terminalia and kept in separate cages until sexual maturity (3 days post eclosion) or as required for mating experiments (see below).

### Oviposition experiments

Three-day old virgin females were injected (Nanoject II, Drummond Scientific/Olinto Martelli Srl, Florence, Italy) with 0.69 μg (138 nl, 5 μg/μl double stranded RNAs [dsRNAs, see below]) targeting JNK pathway components (*JNK1, Jun, Fos*, or *puckered [puc]*) or *GFP*, a gene not expressed in these mosquitoes, as a negative control. In experiments in which two genes were targeted simultaneously (e.g. Fig. 2), a mixture of the two *dsRNAs,* in which the concentration of each was 5 μg/μl, was prepared and injected in the same volume (138 nl) as a single dsRNA treatment. Assignment of mosquitoes to different groups was random but groups were not blinded. Using oviposition protocols described previously^12^ females were blood fed 3 days post-injection, and 2 days later (upon completion of egg development), were induced to oviposit developed eggs either by mating (see below) or by injection of exogenous 20E (Sigma, Milan, Italy; see below). For mating-induced oviposition, females were added in groups of 10–20 to cages of 150–200 age-matched males, captured *in copula* and successful mating was certified using fluorescent microscopy to verify the presence of an auto-fluorescent mating plug. Next day, mated females were placed in individual oviposition cups as described previously^12^. For oviposition induced by exogenous 20E, two days post blood feeding, females were injected (138 nl) with 20E (38 mM, equivalent to 2.5 ng) dissolved in H_2_O containing 10%EtOH and 5% DMSO or with a solvent control (the same diluent minus 20E) then next day placed in individual oviposition cups. Since oviposition, in our hands, routinely takes place 2 days after mating or 20E injection^12^, females were checked every day for four days and oviposition was deemed to have occurred if any eggs were detected in the oviposition cup. Females who died before ovipositing and any females who failed to develop eggs were excluded on the assumption that they had not taken a blood meal. Differences between groups in the frequency of oviposition were assessed using a generalized linear model based on the binary outcome ‘oviposition or no oviposition’ by day 4 post mating or 20E injection or day 5 post blood feeding in the case of *dspuc* treatment. ‘Experiment’ was included as a variable in the model and experiments statistically distinguishable from the others were removed as outliers. In the case of mating-induced oviposition this led to the removal of 2 of 11 experiments while in the case of *dspuc*- or 20E-induced oviposition no outliers were identified. The model was used to calculate the odds ratios (OR) of the relative likelihood of oviposition, or failed oviposition, in each group as well as the statistical significance of inter-group differences.

### Microarray experiments and analysis

Using protocols described previously^12^ heads from 15 mated or age-matched virgin control females were dissected in ice-cold PBS at 3 h and 24 h post mating and immediately transferred to Tri Reagent. Four independent biological replicates were performed on different generations of the same mosquito line (G3). Total RNA was recovered by phenol/chloroform extraction, DNase-treated to remove genomic DNA and quantified using a NanoDrop spectrophotometer (Thermofisher). Using a one-color labelling strategy, RNA (100 ng) from each of the four replicates was labelled using a Low Input Quick Amp Labeling kit (Agilent, Stockport, UK) following protocol G4140-90040. Labelled RNA was hybridised to 44-K *An. gambiae* whole genome microarrays (Design ID G2519F-020449). Labelling, hybridization and scanning were performed by the Institute of Genetics and Molecular and Cellular Biology (Illkirch, France).

Microarray datasets were analyzed using the R statistical software environment (version 2.15.0) running the Linear Models for Microarray Data (Limma) package (version 3.14.4)^58^. Dye signals were background-corrected using the normexp method with an offset of 16 and normalized across microarrays using the quantile method. Multiple probes for the same transcript identifier were collapsed to individual genes, and average fold-change results were generated for each unique array identifier. Package ArrayQualityMetrics^59^ was used for quality control of all microarrays before significance was estimated by fitting a linear model to each gene across replicated arrays, applying a contrast matrix of comparisons of interest in the mating-array experiment, and determining an empirical Bayes moderated t statistic. The decideTests function for multiple testing across genes and contrasts (“global strategy”) was also used to classify the related t statistics as up, down, or not significantly regulated. *P* values then were corrected for multiple testing by the Benjamini–Hochberg method^60^. Results were exported as Microsoft Excel files, and transcript identifiers [Ensembl Gene ID (AGAP) numbers or ESTs] with adjusted P < 0.05 were selected for further analysis. When possible, ESTs were identified by using manual submissions of Blastn^61^ to the *An. gambiae* PEST strain genome or were classified as unknown. Gene function annotation was assigned via VectorBase gene description, AnoXcel summary^62^, and/or orthology.

### Western blotting

Three-day-old virgin females were mated with age-matched males, injected with 20E or dsRNAs, as indicated in the figure legends and described above (oviposition experiments). The tissues indicated were dissected from 10 to 15 females in to 25 μl of homogenization buffer [Tris–HCl, 10 mM (pH 7.4); NaCl, 150 mM; EDTA, 5 mM; Triton X-100 0.5%; sodium dodecylsulfate (SDS), 0.1% w/v; sodium orthovanadate 10 mM, sodium iodoacetate, 5 mM; protease inhibitor cocktail (Sigma, prod. code, p8340) 10 μl/ml; phosphatase inhibitor cocktail III (Sigma, prod. code, p0044) 10 μl/ml; sodium fluoride (1 mM)], manually homogenized, clarified by centrifugation (14,000×*g*, 5 min, RT), and denatured by heating (85 °C, 5 min) in 1xLDS-PAGE sample buffer (Thermofisher) containing dithiothreitol (DTT, 5 mM). Proteins were separated over 4–12% gradient Bis–Tris gels (Thermofisher) and transferred to nitrocellulose membranes which were blocked (1 h, RT) in PBS-Tween (PBS containing 0.05% Tween-20) supplemented with 5% w/v bovine serum albumin then incubated (overnight, 4 °C, 1/1,000 dilution in 5% BSA) with primary antibodies (Cell Signaling Inc, Leiden, The Netherlands); rabbit anti-pJNK (RRID:AB_823588) or anti-pERK (RRID:AB_331646). Membranes were washed (4 × 15 min PBS-tween), incubated (1 h, RT, 1/5,000 dilution in PBS-Tween containing 5% non-fat milk) with anti-rabbit HRP-conjugated secondary (RRID:AB_2536530), washed again (4 × 15 min, PBS-Tween), developed using enhanced chemiluminescence (ECL) (Ammersham, Cambridge, UK) and visualised using a Fusion FX chemiluminscence detector (Vilber-Lourmat, Marne-la-Vallée, France). Membranes were then stripped (15 min, RT, ReStore; Thermofisher) and re-probed (1 h, RT, 1/10,000 diluted in PBS-tween with 5% non-fat milk) with rat anti-β-actin (AbCam, Cambridge, UK, RRID:AB_867488) then washed and developed as above using a goat anti-rat-HRP secondary (RRID:AB_228356). Optical density of bands was analyzed using Image J software^63^ using ‘non-saturated’ signals (Fusion FX software) and values for pJNK were normalized against those of actin in the same sample (relative phosphorylation). Differences in relative phosphorylation between groups were assessed using an unpaired, two-tailed Mann–Whitney test. In Supplementary Fig. S1 ‘fold-change’ data for pJNK and pERK (mated over virgin) were tested for deviation from a hypothetical value of 1 using a one sample t test. Full length versions of the cropped blots appearing in the figures are reproduced in Supplementary Information while unprocessed versions of all the blots used to generate this manuscript are available through Mendeley Data (see data availability statement below).

### RNAi

Double-stranded RNA (dsRNA) constructs targeting *puckered* (*puc*, AGAP004353), *JNK1* (AGAP022950), *Jun* (AGAP006386) and *Fos* (AGAP001093) were prepared using established methods described elsewhere^12^. Briefly, PCR primers (see Supplementary Table S2) designed using the E-RNAi webservice (https://www.dkfz.de/signaling/e-rnai3/) were used to generate blunt ended amplicons from *An. gambiae* cDNA which were ligated in to the TOPO 2.1 vector (Thermofisher) and transformed in to Top10 competent E Coli (Thermofisher) by heat shock. Plasmids were purified by midiprep kit from blue/ white selected colonies and the insert verified by sequencing. These plasmids (made available through Adgene: *pCR2.1 JNK1*, #133284, *pCR2.1 puc*, #133285; *pCR2.1 Jun*, #133286, *pCR2.1 Fos*, #133287) were used to generate amplicons from which gene-specific dsRNAs were prepared using a T7 polymerase in vitro transcription kit (T7 Megascript, Thermofisher). DNase1-treated dsRNAs were purified by phenol/chloroform extraction and re-suspended in H_2_O at 10–20 μg/μL. As a negative control a dsRNA targeting *GFP* was prepared in the same way from an *EGFP* plasmid described previously^64^.

### Gene expression analysis by qRT-PCR

Three-day-old females were injected with *dsGFP* or *dsJNK* and two days later mated with age-matched males, as indicated in the figure legend and described above (oviposition experiments). Gene expression was assessed by qRT-PCR as described previously^13^. Briefly, dissected tissues were recovered directly to 10 μl of RNA-later (Ambion), immediately supplemented with 250 μl Tri-reagent and homogenized using a motorized pestle. RNA in clarified supernatants (14,000×*g*, 15 min, 4 °C) was extracted, DNase digested and eluted using Direct-zol RNA miniprep columns (Zymo Research/Euroclone, Milan, Italy) according to the manufacturer’s instructions. Some of this material (0.5–1 μg) was reverse transcribed to cDNA as described in detail elsewhere^12^. Triplicate 5 μl aliquots were analysed using Fast SybrGreen master Mix (Thermofisher) and the forward and reverse primers listed in Supplementary Table S2. Reactions were run on a QuantStudio 3 thermocycler (Thermofisher). For each sample, the mean number of cycles to cross threshold (CT) obtained for a the gene of interest was expressed relative to that of a reference gene (*Ribosomal protein L19*, *Rpl19*; AGAP004422) whose expression is insensitive to mating^13^ and blood feeding^65^ (delta CT). Delta CT values were used to calculate the relative expression of the gene of interest using the formula: relative expression = 2^(−delta CT)^. On occasion the relative expression in one group (e.g. virgin females) was expressed relative to that in another group (e.g. mated females) as fold-change in expression (delta delta CT). Effects of RNAi-mediated *JNK1* depletion on mating-induced expression changes were analyzed by two-way ANOVA using relative expression data (2^-delta CT^). Efficiency of knock down of target genes was calculated using delta delta CT values from *dstarget* vs *dsGFP* controls and analyzed using a one sample t-test for deviation from a hypothetical value of 100%.

### Statistical analysis

All Statistical analyses were performed using Prism 8.0 (GraphPad, La Jolla, USA) except logistic regression analyses which were performed using JMP version 14 (SAS, Cary, USA). Non-parametric methods were used unless the normality of all groups analyzed could be demonstrated using a Kolmogorov–Smirnov test. In all tests, a significance cut-off of *p* = 0.05 was applied. Comparisons of multiple treatment groups to a single ‘control’ group were corrected using Dunnett’s (for parametric tests) or Dunn’s (for non-parametric tests) multiple comparison correction. If all possible intergroup comparisons were made, Tukey’s correction was used. On occasion (Supplementary Figs. S1, S2) the effect of a manipulation was assessed using a one sample t-test. In these cases, the normality of all groups was verified using a Kolmogorov–Smirnov test (Supplementary Fig. S2) or a Shapiro–Wilk test (Supplementary Fig. S1).

## Supplementary information

Supplementary information

## Data Availability

Plasmids encoding *dsRNAs* generated in this manuscript are publicly available through Adgene. The microarray data reported are available through ArrayExpress (E-MTAB-8733). Other raw data reported here is available through the Mendeley Data repository: https://data.mendeley.com/datasets/5gmsnv8zw9/2. A preprint version of this manuscript is available at bioRxiv: https://biorxiv.org/cgi/content/short/2020.03.14.990945v1.

## References

[1] WHO (2019). World Malaria Report 2019.

[2] Hemingway J (2014). The role of vector control in stopping the transmission of malaria: Threats and opportunities. Philos. Trans. R Soc. Lond. B Biol. Sci..

[3] Childs LM (2016). Disrupting mosquito reproduction and parasite development for malaria control. PLoS Pathog..

[4] Hammond A (2016). A CRISPR-Cas9 gene drive system targeting female reproduction in the malaria mosquito vector *Anopheles gambiae*. Nat. Biotechnol..

[5] Mitchell SN, Catteruccia F (2017). Anopheline reproductive biology: Impacts on vectorial capacity and potential avenues for malaria control. Cold Spring Harb. Perspect. Med..

[6] Baldini F, Gabrieli P, Rogers DW, Catteruccia F (2012). Function and composition of male accessory gland secretions in *Anopheles gambiae*: A comparison with other insect vectors of infectious diseases. Pathog. Glob. Health.

[7] Rogers DW (2009). Transglutaminase-mediated semen coagulation controls sperm storage in the malaria mosquito. PLoS Biol..

[8] Pondeville E, Maria A, Jacques JC, Bourgouin C, Dauphin-Villemant C (2008). *Anopheles gambiae* males produce and transfer the vitellogenic steroid hormone 20-hydroxyecdysone to females during mating. Proc. Natl. Acad. Sci. USA.

[9] Baldini F (2013). The interaction between a sexually transferred steroid hormone and a female protein regulates oogenesis in the malaria mosquito *Anopheles gambiae*. PLoS Biol..

[10] Mitchell SN (2015). Mosquito biology. Evolution of sexual traits influencing vectorial capacity in anopheline mosquitoes. Science.

[11] Pondeville E (2019). Evolution of sexually-transferred steroids and mating-induced phenotypes in *Anopheles mosquitoes*. Sci. Rep..

[12] Gabrieli P (2014). Sexual transfer of the steroid hormone 20E induces the postmating switch in *Anopheles gambiae*. Proc. Natl. Acad. Sci. USA.

[13] Shaw WR (2014). Mating activates the heme peroxidase HPX15 in the sperm storage organ to ensure fertility in *Anopheles gambiae*. Proc. Natl. Acad. Sci. USA.

[14] Ishimoto H, Kitamoto T (2011). Beyond molting–roles of the steroid molting hormone ecdysone in regulation of memory and sleep in adult Drosophila. Fly (Austin).

[15] Ishimoto H, Sakai T, Kitamoto T (2009). Ecdysone signaling regulates the formation of long-term courtship memory in adult *Drosophila melanogaster*. Proc. Natl. Acad. Sci. USA.

[16] Rubinstein CD, Wolfner MF (2013). Drosophila seminal protein ovulin mediates ovulation through female octopamine neuronal signaling. Proc. Natl. Acad. Sci. USA.

[17] Chen PS (1988). A male accessory gland peptide that regulates reproductive behavior of female *D. melanogaster*. Cell.

[18] Yapici N, Kim YJ, Ribeiro C, Dickson BJ (2008). A receptor that mediates the post-mating switch in Drosophila reproductive behaviour. Nature.

[19] Wang F (2020). Neural circuitry linking mating and egg laying in Drosophila females. Nature.

[20] Haussmann IU, Hemani Y, Wijesekera T, Dauwalder B, Soller M (2013). Multiple pathways mediate the sex-peptide-regulated switch in female Drosophila reproductive behaviours. Proc. Biol. Sci..

[21] Dalton JE (2010). Dynamic, mating-induced gene expression changes in female head and brain tissues of *Drosophila melanogaster*. BMC Genom..

[22] Wang MC, Bohmann D, Jasper H (2005). JNK extends life span and limits growth by antagonizing cellular and organism-wide responses to insulin signaling. Cell.

[23] Wang MC, Bohmann D, Jasper H (2003). JNK signaling confers tolerance to oxidative stress and extends lifespan in Drosophila. Dev. Cell.

[24] Garver LS, de Almeida Oliveira G, Barillas-Mury C (2013). The JNK pathway is a key mediator of *Anopheles gambiae* antiplasmodial immunity. PLoS Pathog..

[25] Ramphul UN, Garver LS, Molina-Cruz A, Canepa GE, Barillas-Mury C (2015). Plasmodium falciparum evades mosquito immunity by disrupting JNK-mediated apoptosis of invaded midgut cells. Proc. Natl. Acad. Sci. USA.

[26] Rogers DW (2008). Molecular and cellular components of the mating machinery in *Anopheles gambiae* females. Proc. Natl. Acad. Sci. USA.

[27] Nsango SE (2013). AP-1/Fos-TGase2 axis mediates wounding-induced *Plasmodium falciparum* killing in *Anopheles gambiae*. J. Biol. Chem..

[28] Barillas-Mury C (2007). CLIP proteases and Plasmodium melanization in *Anopheles gambiae*. Trends Parasitol..

[29] Michel K, Kafatos FC (2005). Mosquito immunity against Plasmodium. Insect Biochem. Mol. Biol..

[30] Lee WJ, Miura M (2014). Mechanisms of systemic wound response in Drosophila. Curr. Top. Dev. Biol..

[31] Bidla G, Hauling T, Dushay MS, Theopold U (2009). Activation of insect phenoloxidase after injury: Endogenous versus foreign elicitors. J. Innate Immun..

[32] Pinto SB (2009). Discovery of Plasmodium modulators by genome-wide analysis of circulating hemocytes in *Anopheles gambiae*. Proc. Natl. Acad. Sci. USA.

[33] Fogarty CE (2016). Extracellular reactive oxygen species drive apoptosis-induced proliferation via Drosophila macrophages. Curr. Biol..

[34] Stramer B (2008). Gene induction following wounding of wild-type versus macrophage-deficient *Drosophila embryos*. EMBO Rep..

[35] Christensen BM, Li J, Chen CC, Nappi AJ (2005). Melanization immune responses in mosquito vectors. Trends Parasitol..

[36] Tang H (2009). Regulation and function of the melanization reaction in Drosophila. Fly (Austin).

[37] Blandin SA, Marois E, Levashina EA (2008). Antimalarial responses in *Anopheles gambiae*: From a complement-like protein to a complement-like pathway. Cell Host Microbe.

[38] Povelones M, Upton LM, Sala KA, Christophides GK (2011). Structure-function analysis of the *Anopheles gambiae* LRIM1/APL1C complex and its interaction with complement C3-like protein TEP1. PLoS Pathog..

[39] Fraiture M (2009). Two mosquito LRR proteins function as complement control factors in the TEP1-mediated killing of Plasmodium. Cell Host Microbe.

[40] Blandin S (2004). Complement-like protein TEP1 is a determinant of vectorial capacity in the malaria vector *Anopheles gambiae*. Cell.

[41] Ramet M, Lanot R, Zachary D, Manfruelli P (2002). JNK signaling pathway is required for efficient wound healing in Drosophila. Dev. Biol..

[42] Souvannaseng L (2018). Inhibition of JNK signaling in the Asian malaria vector *Anopheles stephensi* extends mosquito longevity and improves resistance to *Plasmodium falciparum* infection. PLoS Pathog..

[43] Krishna M, Narang H (2008). The complexity of mitogen-activated protein kinases (MAPKs) made simple. Cell Mol Life Sci.

[44] Martin-Blanco E (1998). puckered encodes a phosphatase that mediates a feedback loop regulating JNK activity during dorsal closure in Drosophila. Genes Dev..

[45] Mack PD, Kapelnikov A, Heifetz Y, Bender M (2006). Mating-responsive genes in reproductive tissues of female *Drosophila melanogaster*. Proc. Natl. Acad. Sci. USA.

[46] Mattei AL, Riccio ML, Avila FW, Wolfner MF (2015). Integrated 3D view of postmating responses by the *Drosophila melanogaster* female reproductive tract, obtained by micro-computed tomography scanning. Proc. Natl. Acad. Sci. USA.

[47] Canyon DV, Hii JL, Muller R (1999). Adaptation of *Aedes aegypti* (Diptera: Culicidae) oviposition behavior in response to humidity and diet. J. Insect Physiol..

[48] Sylvestre G, Gandini M, Maciel-de-Freitas R (2013). Age-dependent effects of oral infection with dengue virus on *Aedes aegypti* (Diptera: Culicidae) feeding behavior, survival, oviposition success and fecundity. PLoS One.

[49] Shaw WR (2016). Wolbachia infections in natural Anopheles populations affect egg laying and negatively correlate with Plasmodium development. Nat. Commun..

[50] Nepomichene TN, Andrianaivolambo L, Boyer S, Bourgouin C (2017). Efficient method for establishing F1 progeny from wild populations of Anopheles mosquitoes. Malar. J..

[51] Hirashima A, Rauschenbach I, Sukhanova M (2000). Ecdysteroids in stress responsive and nonresponsive *Drosophila virilis* lines under stress conditions. Biosci. Biotechnol. Biochem..

[52] Zheng W (2018). Dehydration triggers ecdysone-mediated recognition-protein priming and elevated anti-bacterial immune responses in Drosophila Malpighian tubule renal cells. BMC Biol..

[53] Ishimoto H, Kitamoto T (2010). The steroid molting hormone Ecdysone regulates sleep in adult *Drosophila melanogaster*. Genetics.

[54] Parducz A (2006). Synaptic remodeling induced by gonadal hormones: Neuronal plasticity as a mediator of neuroendocrine and behavioral responses to steroids. Neuroscience.

[55] Lehmann M, Jiang C, Ip YT, Thummel CS (2002). AP-1, but not NF-kappa B, is required for efficient steroid-triggered cell death in Drosophila. Cell Death Differ..

[56] Zhu S, Chen R, Soba P, Jan YN (2019). JNK signaling coordinates with ecdysone signaling to promote pruning of Drosophila sensory neuron dendrites. Development.

[57] Werling K (2019). Steroid hormone function controls non-competitive plasmodium development in Anopheles. Cell.

[58] Smyth GK, Michaud J, Scott HS (2005). Use of within-array replicate spots for assessing differential expression in microarray experiments. Bioinformatics.

[59] Kauffmann A, Gentleman R, Huber W (2009). arrayQualityMetrics—a bioconductor package for quality assessment of microarray data. Bioinformatics.

[60] Benjamini Y, Hochberg Y (1995). Controlling the false discovery rate: A practical and powerful approach to multiple testing. J. R. Stat. Soc. Ser. B.

[61] Altschul SF, Gish W, Miller W, Myers EW, Lipman DJ (1990). Basic local alignment search tool. J. Mol. Biol..

[62] Ribeiro JM, Topalis P, Louis C (2004). AnoXcel: An *Anopheles gambiae* protein database. Insect. Mol. Biol..

[63] Schneider CA, Rasband WS, Eliceiri KW (2012). NIH Image to ImageJ: 25 years of image analysis. Nat. Methods.

[64] Marois E (2012). High-throughput sorting of mosquito larvae for laboratory studies and for future vector control interventions. Malar. J..

[65] Marinotti O, Nguyen QK, Calvo E, James AA, Ribeiro JM (2005). Microarray analysis of genes showing variable expression following a blood meal in *Anopheles gambiae*. Insect Mol. Biol..

