# Supplementary material for: JNK signaling regulates oviposition in the malaria vector Anopheles gambiae
Source: Sci Rep. 2020 Sep 1;10:14344. doi: 10.1038/s41598-020-71291-5 (PMC7462981; doi:10.1038/s41598-020-71291-5)

**SUPPLEMENTARY INFORMATION**

**JNK signaling regulates oviposition in the malaria vector *Anopheles gambiae***

Matthew J. Peirce, Sara N. Mitchell, Evdoxia G. Kakani, Paolo Scarpelli, Adam South, W. Robert Shaw, Kristine L. Werling, Paolo Gabrieli, Perrine Marcenac, Martina Bordoni, Vincenzo Talesa and Flaminia Catteruccia


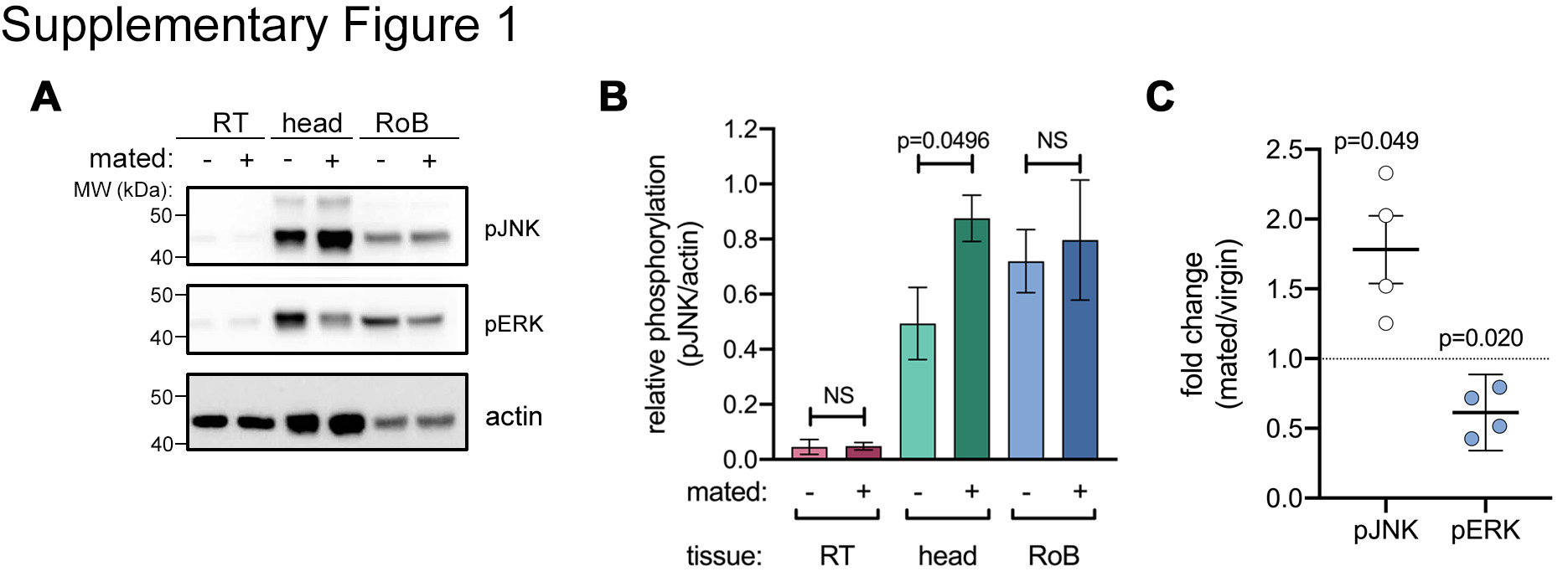


**Supplementary Figure 1. Mating induces pJNK selectively in the head. A.** Representative western blot of reproductive tracts (RT; ovaries, atrium and spermatheca), heads or rest of the body (RoB) prepared from 3-day-old virgin (-) or mated (+) females (10-15 pooled tissues per point) 2 hours after mating. In **B** the optical density of bands was quantified (ImageJ) and the pJNK signal was normalized against actin and expressed as ‘relative phosphorylation’. Data represent the mean ± SEM for four (RT, head) or three (RoB) independent biological replicates. Differences in the relative pJNK levels in virgin and mated tissues were analyzed using a two-tailed t test and significant *p* values (*p*<0.05) reported, ns denotes ‘not significant’ (*p*>0.05). In **C** the data represent the mating-induced fold change (mean ± 95% confidence interval) in actin-normalized pJNK (white circles) and pERK signals (blue circles) in the head in four independent biological replicates. Fold-change values were compared against a hypothetical value of 1 (indicated by a dotted line) using a one sample t-test. Exact *p* values are given and *p*<0.05 was taken to be significant. The t statistic and degrees for freedom were: pJNK*,*  t=3.22, df=3; pERK, t=4.51, df=3. Both groups passed a Shapiro-Wilk normality test (pJNK*, p*=0.77; pERK*, p*=0.59).


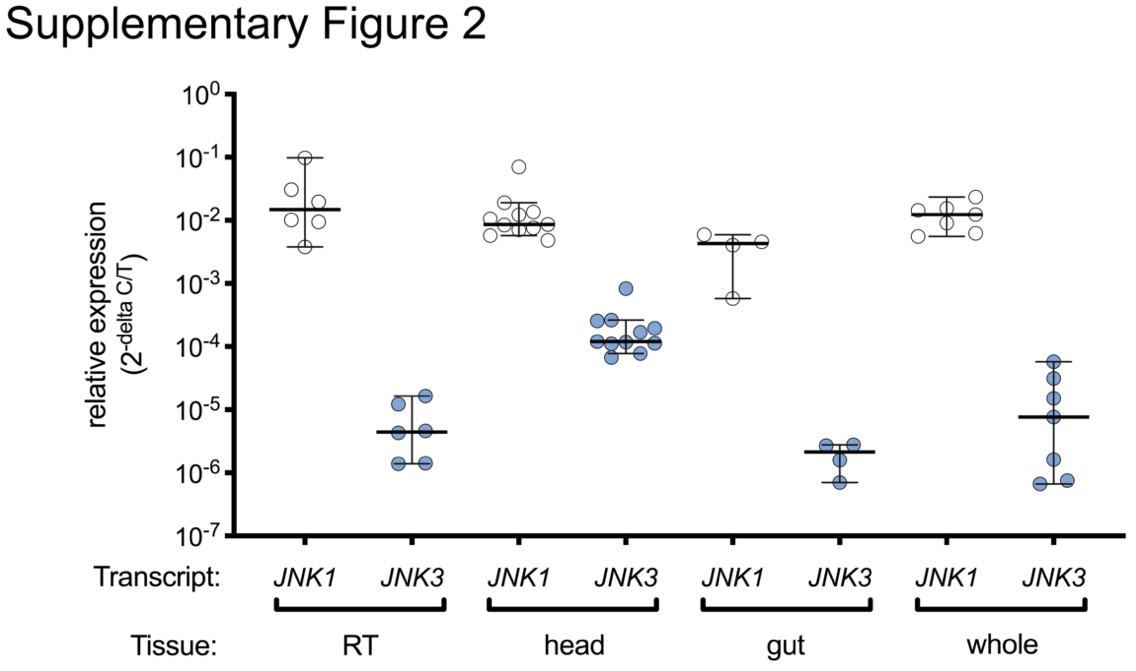


**Supplementary Figure 2. Relative expression of *JNK1* and *JNK3* in *An. gambiae* tissues.** The expression of transcripts for *JNK1* (AGAP029555, white circles) and *JNK3* (AGAP009460, blue circles) was measured by qRT-PCR and expressed relative to *Rpl19*, a loading control (delta C/T), in the indicated tissues or from ‘whole’ virgin 3-day-old females. Each point represents an independent biological replicate comprising a pool of 5-10 tissues/ sample. Bars represent the median ± 95% confidence interval of values detected.


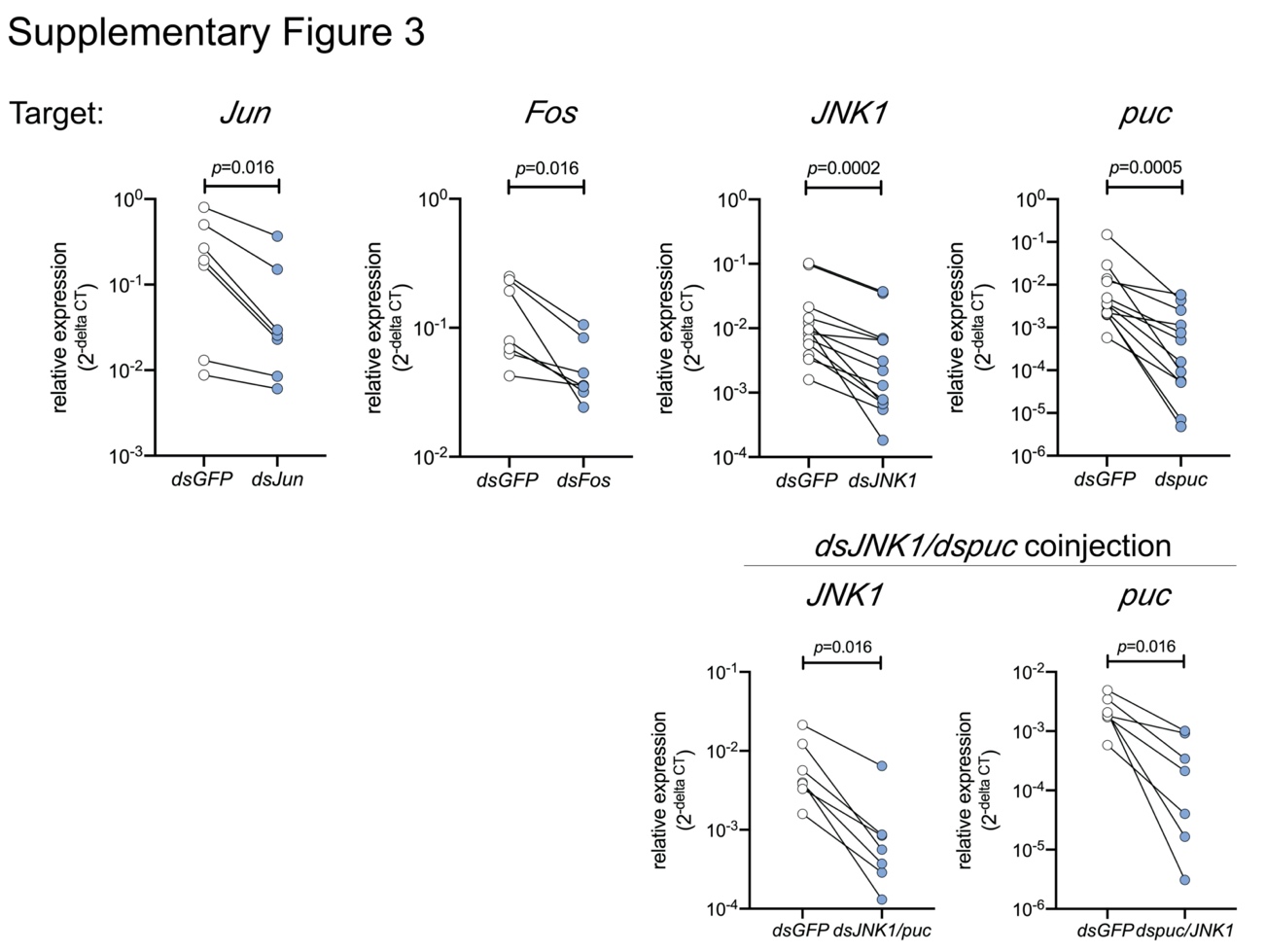


**Supplementary Figure 3. RNAi-mediated knock down of JNK pathway components with *dsRNAs*.** The effect of single or combined *dsRNA* treatments on the target gene indicated was measured in a pool of 4-6 whole females, two days (*JNK1, puc, joint JNK1/puc*) or five days (*Jun, Fos*) after injection. The data presented show the *Rpl19*-normalized relative expression values (2^-delta CT) in *dsGFP*-injected controls (white circles) and *dstarget*-injected females (blue circles) for each gene analysed. Each pair of connected dots represents an independent biological replicate (different treatments of pooled mosquitoes from the same generation). Differences between *dstarget*- and *dsGFP*-treated relative expression values were analyzed using a Wilcoxon signed rank test and exact *p* values reported. *p*<0.05 was taken to be significant. The mean ± SEM knock down efficiencies were: *dsJun* 65±9% (n=7); *dsFos* 53±8% (n=7); *dsJNK1* 69±5% (n=13), *dspuc* 86±5% (n=12). Coinjection of *dsJNK* and *dspuc* effectively reduced expression of both *JNK1* (85±4%, n=7) and *puc* (85±7%, n=7).


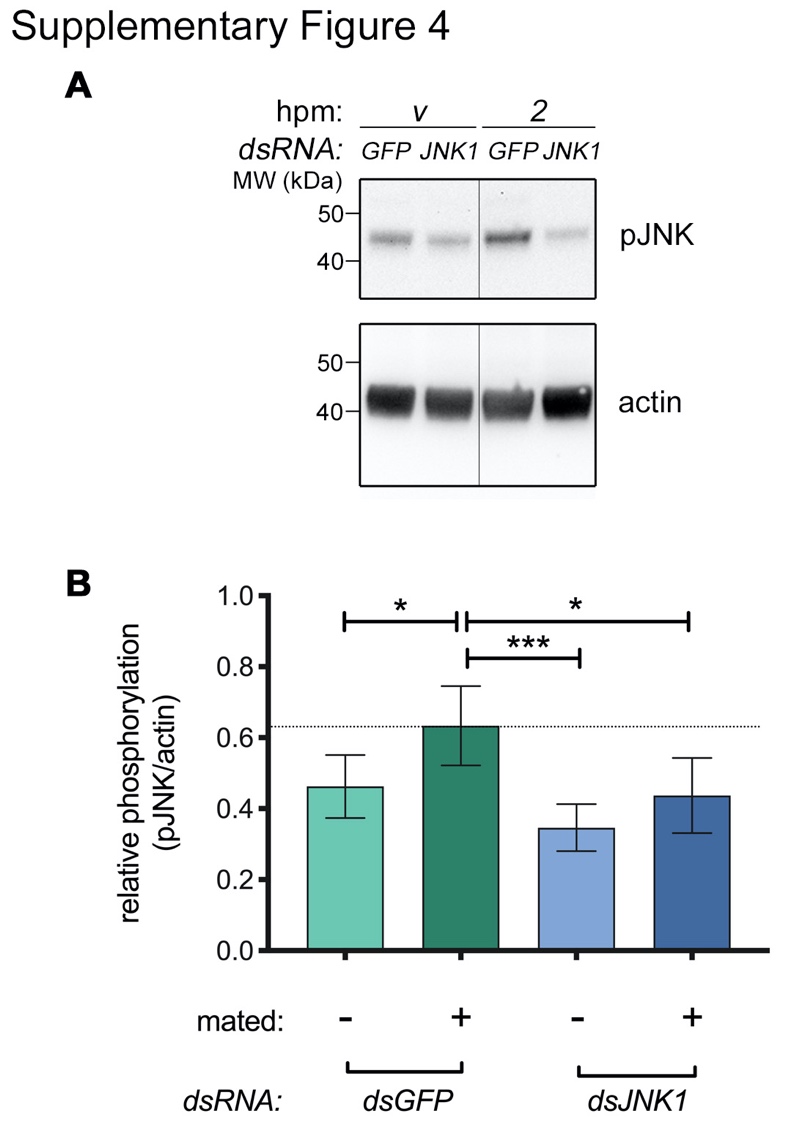


**Supplementary Figure 4. *dsJNK1* inhibits mating-induced increase in pJNK in the head.** **A.** Representative western blot of heads dissected from 10-15 *dsGFP-* or *dsJNK1*-injected virgin (v) or mated females at 2 hours post mating (hpm). Samples were western blotted using anti-pJNK then stripped and re-probed with anti-actin as loading control. The optical density of bands was quantified (ImageJ), the pJNK signal normalized against actin and expressed as ‘relative phosphorylation’. The line separating the virgin and 2hpm time points indicates the removal of an irrelevant intervening lane. Data in panel **B** represent the mean ± SEM of 5 similar experiments. Inter-group differences were compared using a 2-way ANOVA test with Tukey’s multiple comparison correction. All statistically significant (*p*<0.05) differences are indicated: * denotes *p*<0.05, *** denotes *p*<0.005.


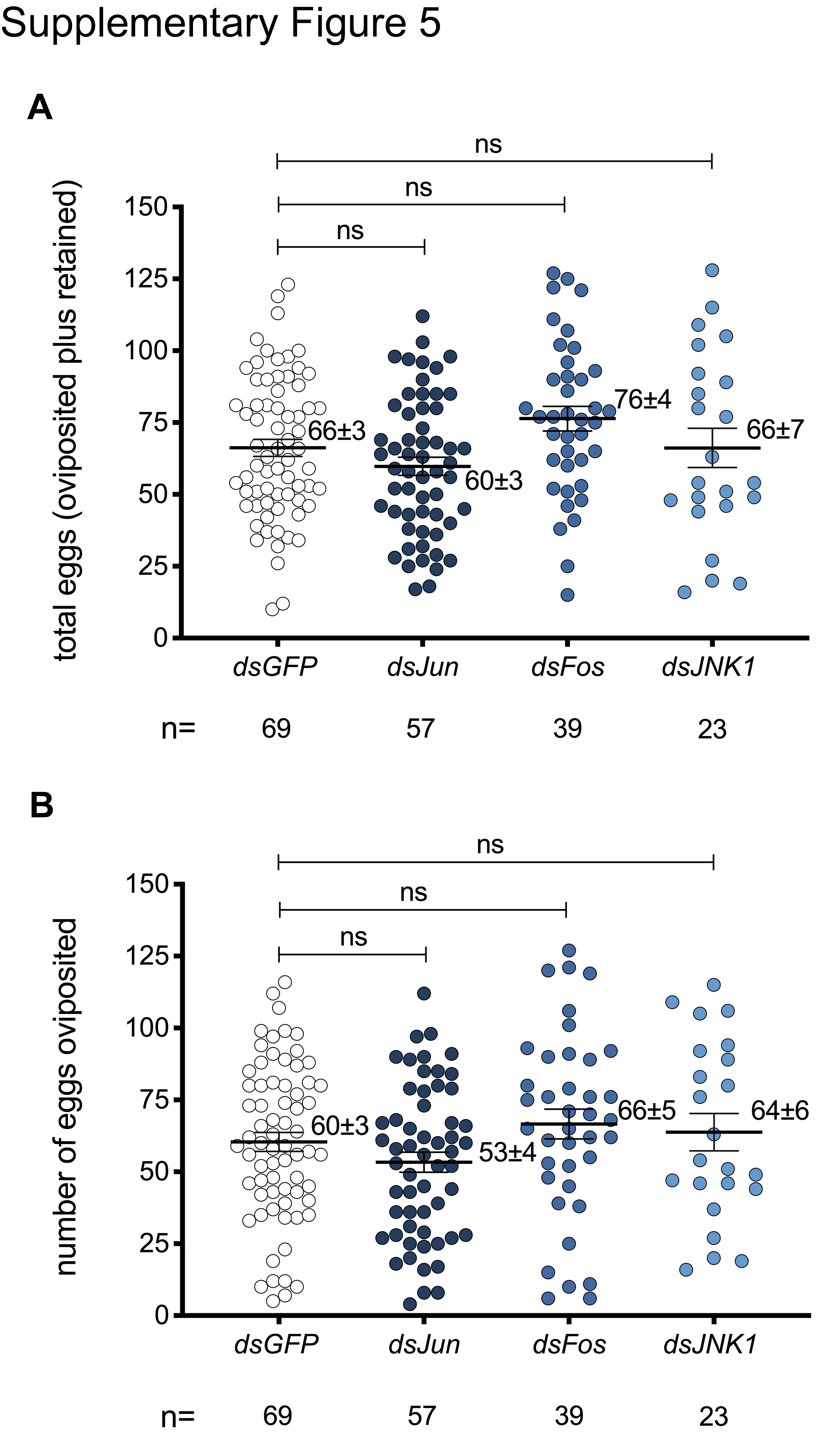


**Supplementary Figure 5. JNK pathway depletion has no effect on number of eggs developed.** Virgin females were injected with the indicated *dsRNAs* and blood-fed 3 days later. After completion of egg development (2 days post blood feeding), females were mated to induce oviposition. The total number of eggs developed (the number of eggs oviposited plus the number of eggs retained in the abdominal cavity, **A**) and the number of eggs oviposited (**B**) were counted in those females who oviposited 4 days post blood feeding. The data presented represent the mean ± SEM of five, independent biological replicates. Using a one-way ANOVA test with Dunnett’s multiple comparison correction all differences between control (*dsGFP*) and treatment groups were ‘not significant’ (ns, corrected p values >0.05).


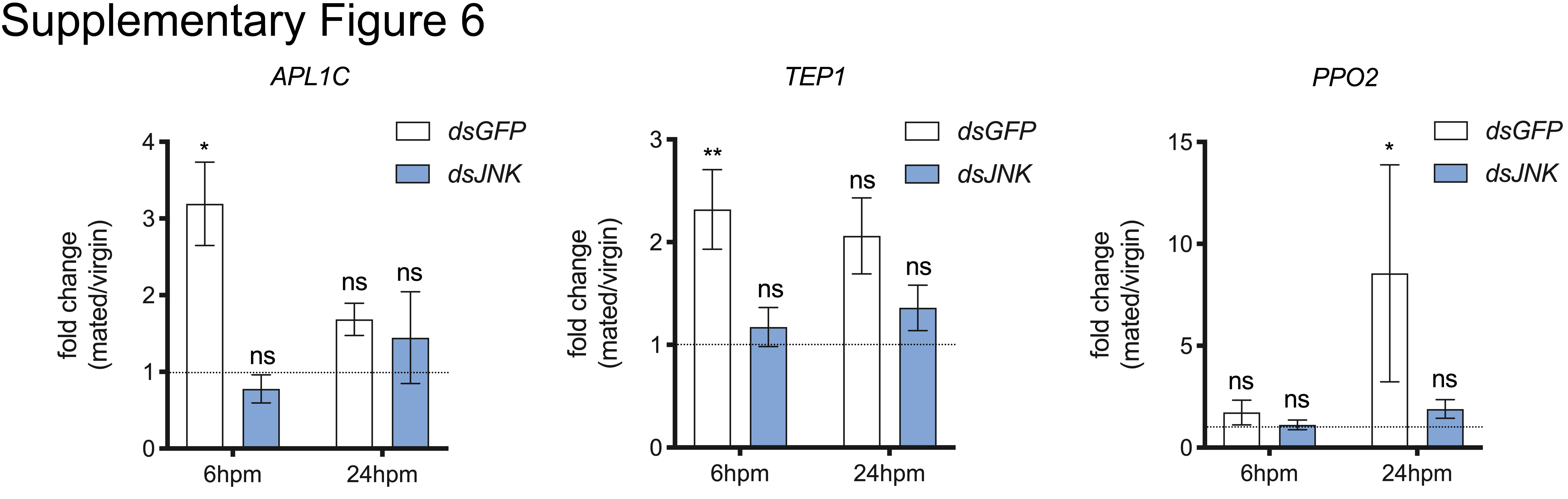


**Supplementary Figure 6. Mating- and JNK1-dependence of genes expressed in the head.** Heads of 5-10 *dsGFP-* (control) or *dsJNK1-* injected females were dissected at 6 and 24 hours after mating or at the same time points from age-matched virgin controls, and analyzed by qRT-PCR for the gene of interest indicated. The data presented represent the mean ± SEM fold expression change in mated females relative to age-matched virgins of the same group (1, indicated by a dotted line) and comprise at least 6 independent biological replicates. Inter-group differences at a given time point were assessed by comparing relative expression (delta CT) in the *dsGFP* virgin control group to all other treatments using a 2-way ANOVA test with Dunnett’s multiple comparison correction. Statistical significance was ascribed to *p*<0.05: * denotes *p*<0.05; ** denotes *p*<0.01; ns denotes ‘not significant’ (*p*>0.05).

**Supplementary Table 1. Low frequency of oviposition following vehicle control injection**

| **Treatment** | **Oviposition Frequency** |
| --- | --- |
| *dsGFP* alone (Fig. 2d) | 1.7%, n=54 |
| *dsJNK1* alone (Fig. 2d) | 3.1%, n=48 |
| *dsGFP* then 20E (Fig. 3d) | 76.2%, n=101 |
| *dsGFP* then solvent | 3.0%, n=67 |
| *dsJNK1* then solvent | 0%, n=26 |
| *dsJun* then solvent | 4.8%, n=62 |
| *dsFos* then solvent | 5.7%, n=35 |

**Supplementary Table 1. Low frequency of oviposition following vehicle control injection.** Virgin females used in Figure 3c were injected with dsRNAs, blood-fed 3 days post-injection then 2 days later injected with solvent control (5% DMSO, 10% EtOH in H_2_O) and placed in oviposition cups. The data presented represent data pooled from three or more independent biological replicates and show the number of females injected (n) and the percentage ovipositing at least one egg by day 4 post injection.

**Supplementary Table 2. Primers used in the preparation of *dsRNAs* and in qRT-PCR experiments**

| **dsRNA** | **Fwd** | **Rev** |
| --- | --- | --- |
| ***JNK1 (AGAP029555)*** | GTCACGCCTTCTACACCGTC | CAGCCCAAAGTCGAGGATT |
| ***Jun (AGAP006386)*** | CCACCCTCGAGCTGAACCTCGG | GTGGTGTTGGTGGGCACGTTCG |
| ***Fos (AGAP001093)*** | TATGATGCGCAGTGCATTTG | CGTGACTTGTGCTGATAACG |
| ***Puc (AGAP004353)*** | TGAAACATAAATCGCCCTGC | GACAGACCCTTGTAGCGCAT |
| ***GFP*** | TGTTCTGCTGGTAGTGGTCG | ACGTAAACGGCCACAAGTTC |
| ***T7*** | taatacgactcactatagggCCGCCAGTG  TGCTGGAA | taatacgactcactatagggCCAGTGTGAT  GGATATCTGCAGAA |
| **qRT-PCR** |  |  |
| ***APL1C (AGAP007033)*** | CAATGTGCTGGTTACACGCC | CCACATGTGAAGAAAATCACACTGA |
| ***PPO2 (AGAP006258)*** | GAACTTGACCCTAACGCACC | GTCCGGATACTTCTTGTCGC |
| ***TEP1 (AGAP010815)*** | CTCATGGGCTACTGGTTGT | GCTGTGAGTTAAAGTTGCTGAT |
| ***Rpl19 (AGAP004422)*** | CCAACTCGCGACAAAACATTC | ACCGGCTTCTTGATGATCAGA |
| ***Puc*** | GCCTAGTGGTCAGCTGAAGC | GCCTCGATCAGCGACAGACC |
| ***Fos*** | CAACGCTCCCGTTCAACCG | GTTGGCGTAGTACGTGTCGGC |
| ***Jun*** | AGGGCAAGTTTTGAATGCAC | CACGCACTTTCTCCCTTTGT |
| ***JNK1*** | GCCGAAGAACGACAAACTATGTGC | GCTGTGTTACTGTATCGTATGCG |

|  |
| --- |

**Supplementary Table 2. *dsRNA* and qRT-PCR primers used in this study.** All qRT-PCR primers were used at 300nM except *Rpl19* Rev which was used at 900nM. PCR products for dsRNA generation were all prepared using the listed primers at 200nM.

**FULL LENGTH WESTERN BLOTS**

**Peirce et al Figure 1a and 3a**

pJNK (head)


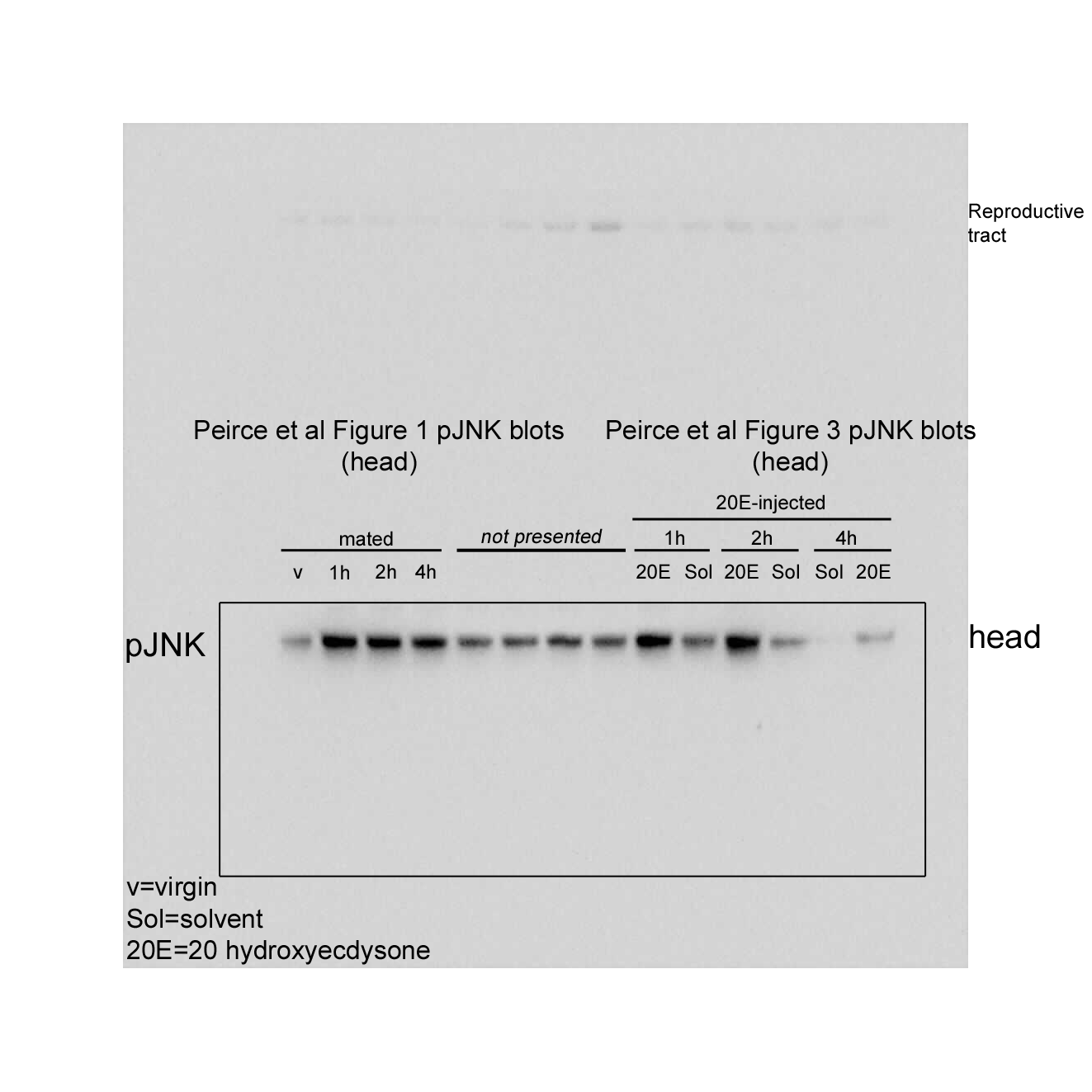


**Peirce et al Figure 1c and 3c**

pJNK (reproductive tract)


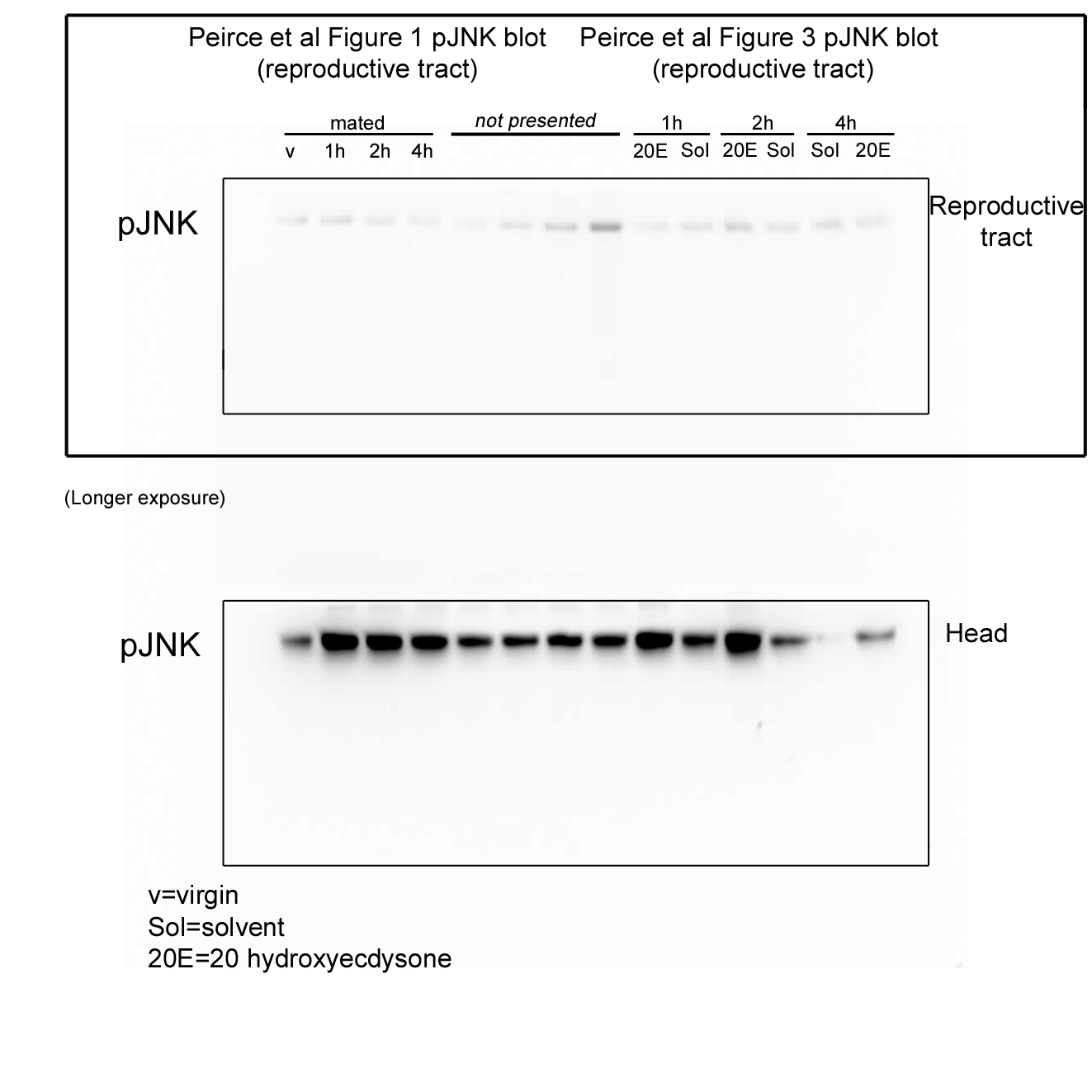


**Peirce et al Figure 1a, c and 3a, c**

Actin re-probes (head and reproductive tract)


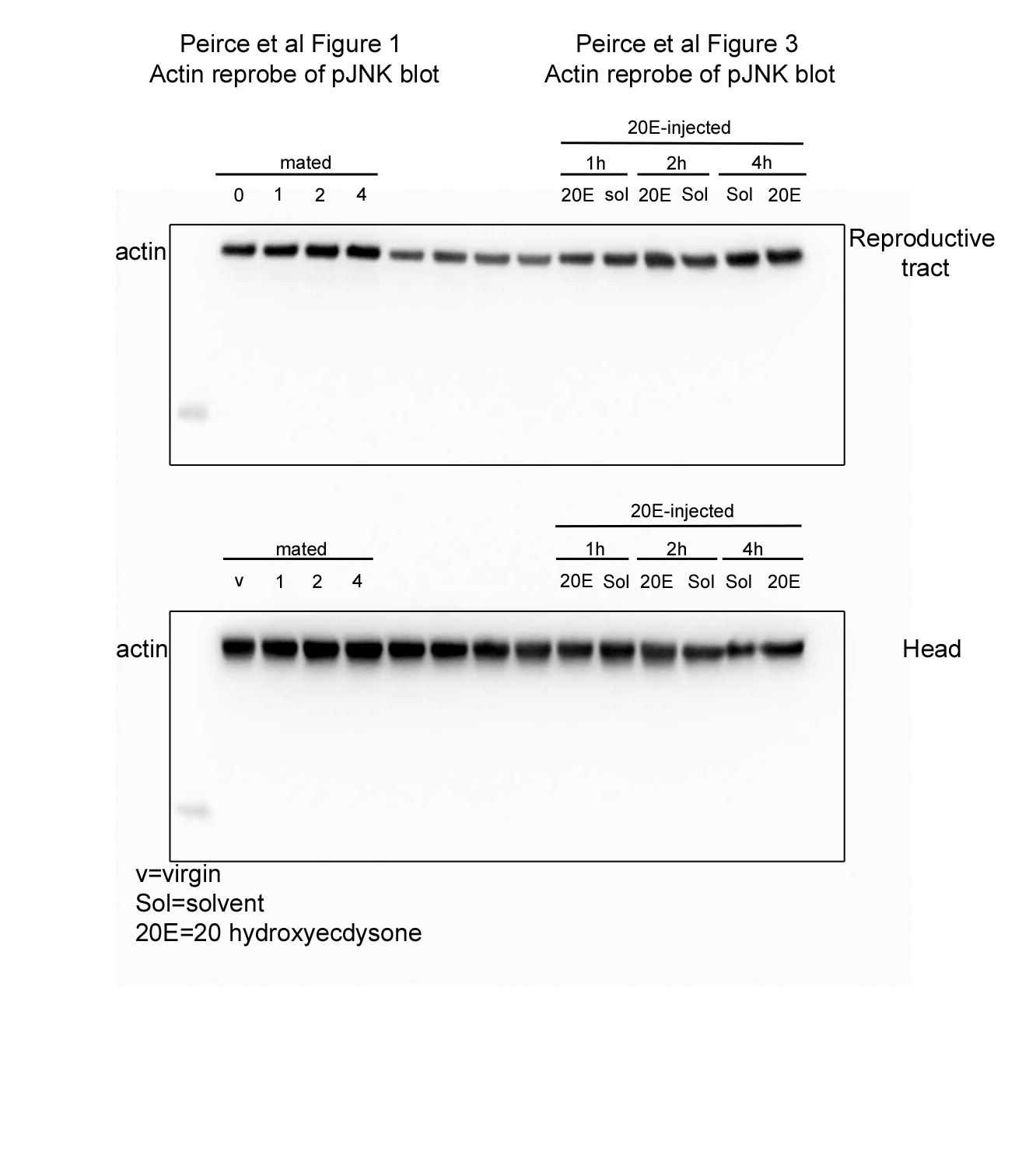


**Peirce et al Figure 1a, c and 3a, c**

Molecular weight ladder


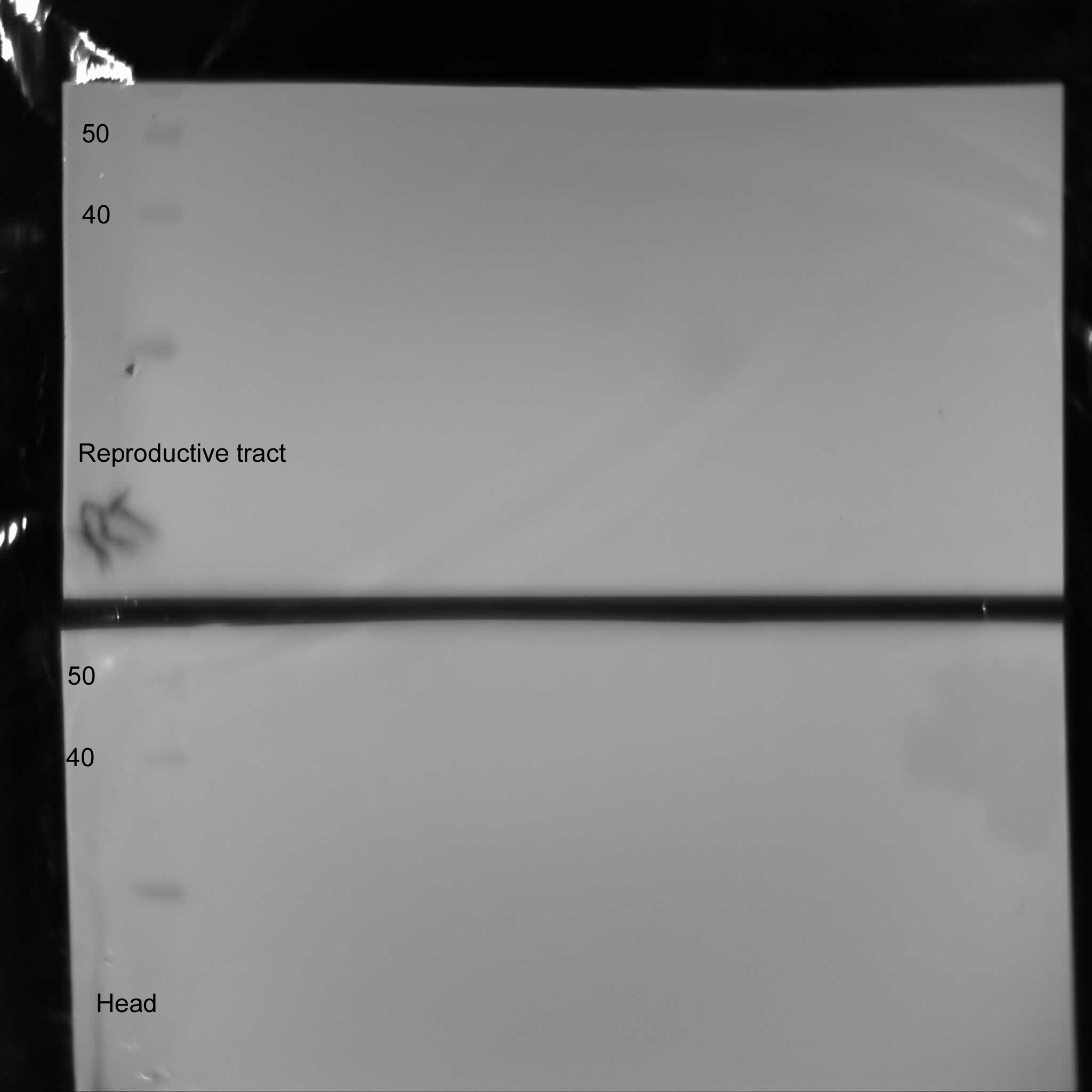


**Peirce et al Figure 2a**

pJNK (head)


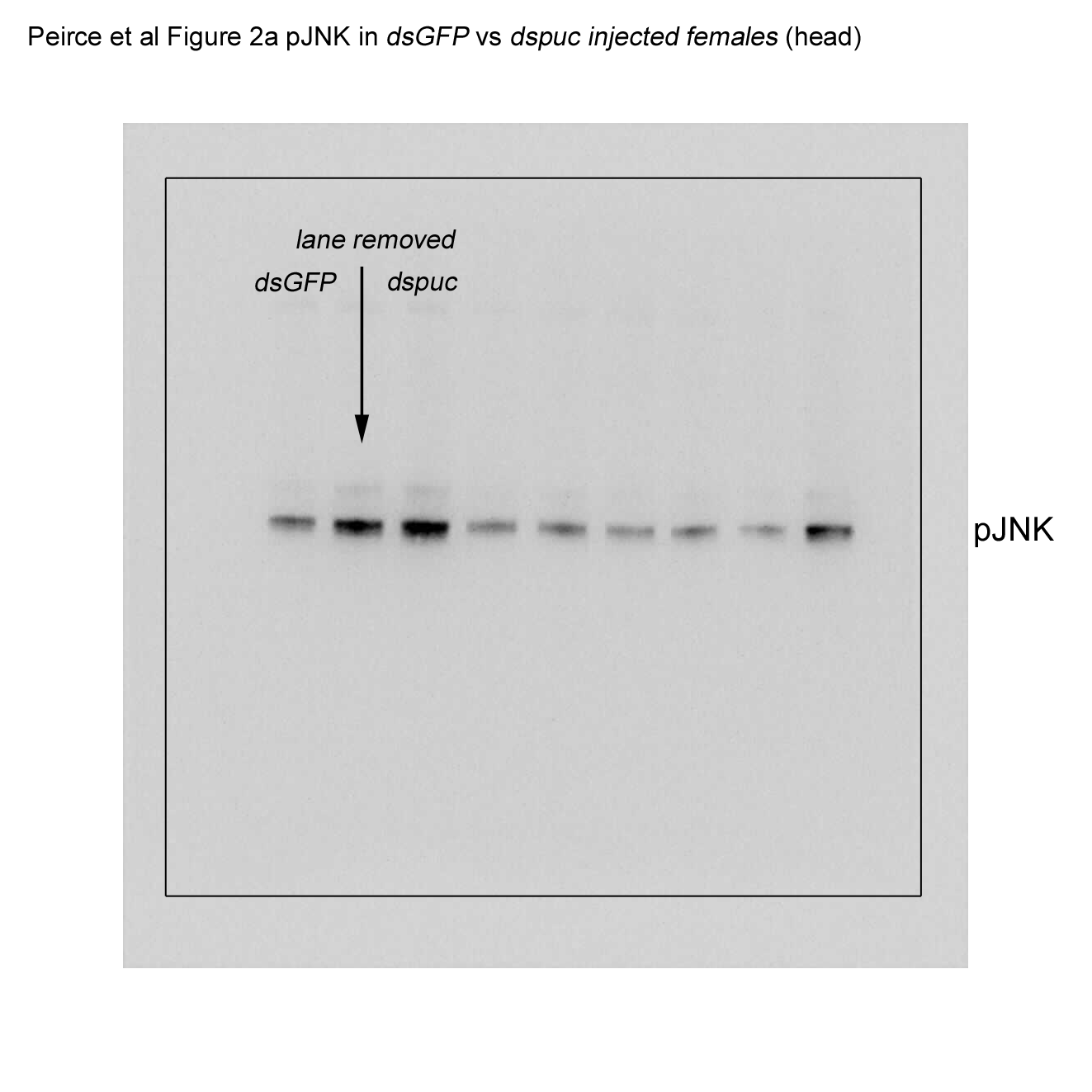


**Peirce et al Figure 2a**

Actin re-probe (head)


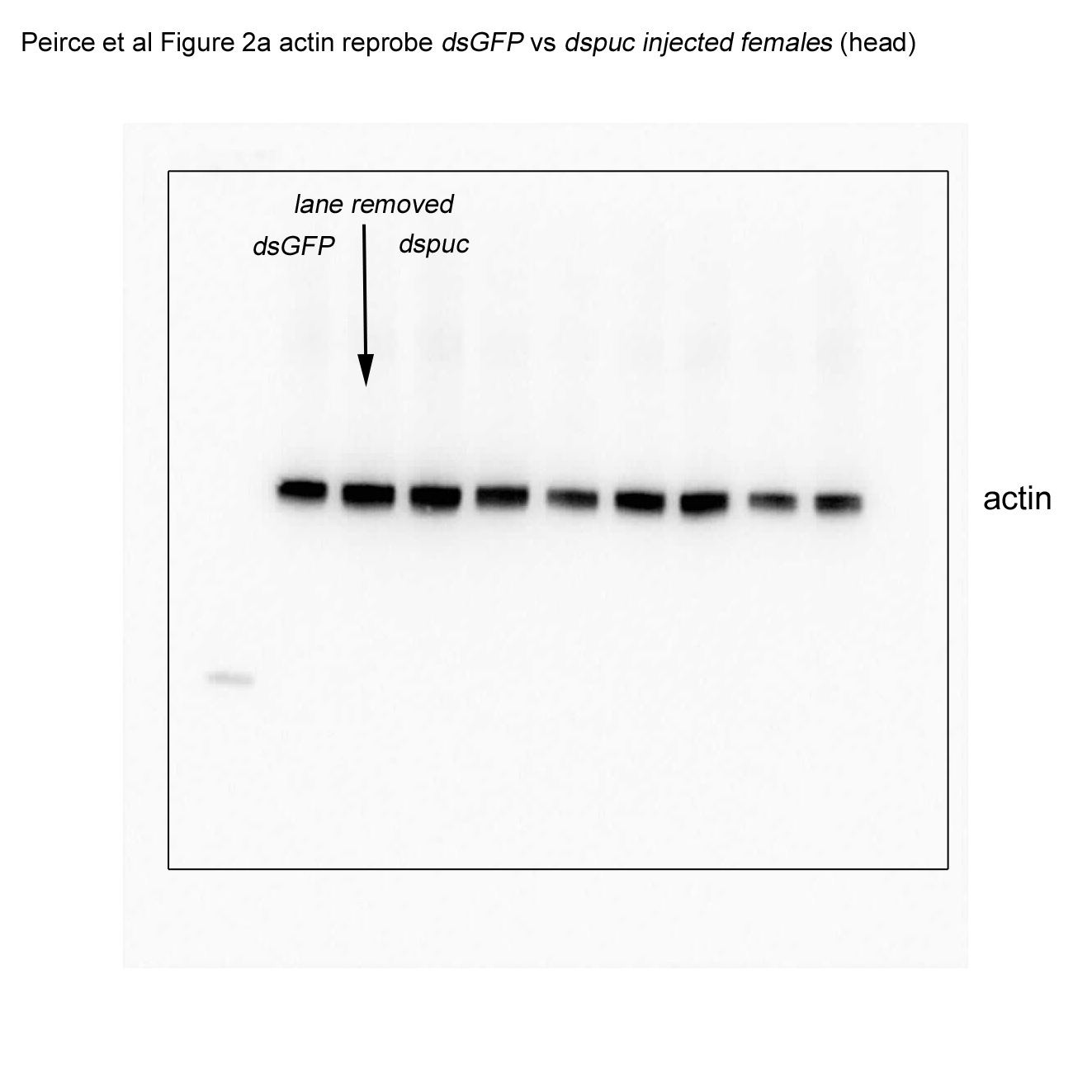


**Peirce et al Figure 2a**

molecular weight ladder (head)


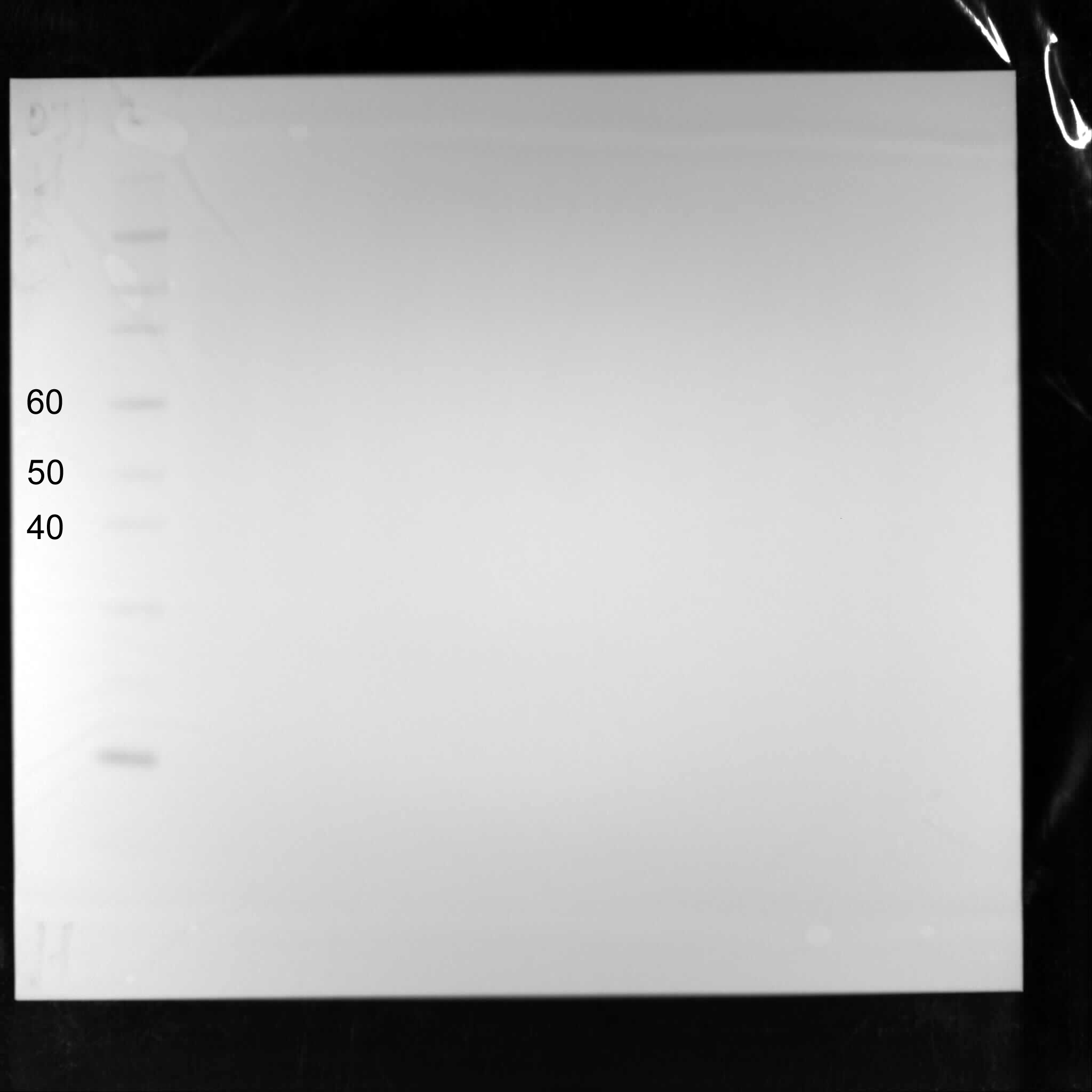


**Peirce et al Figure 2c**

pJNK (reproductive tract)


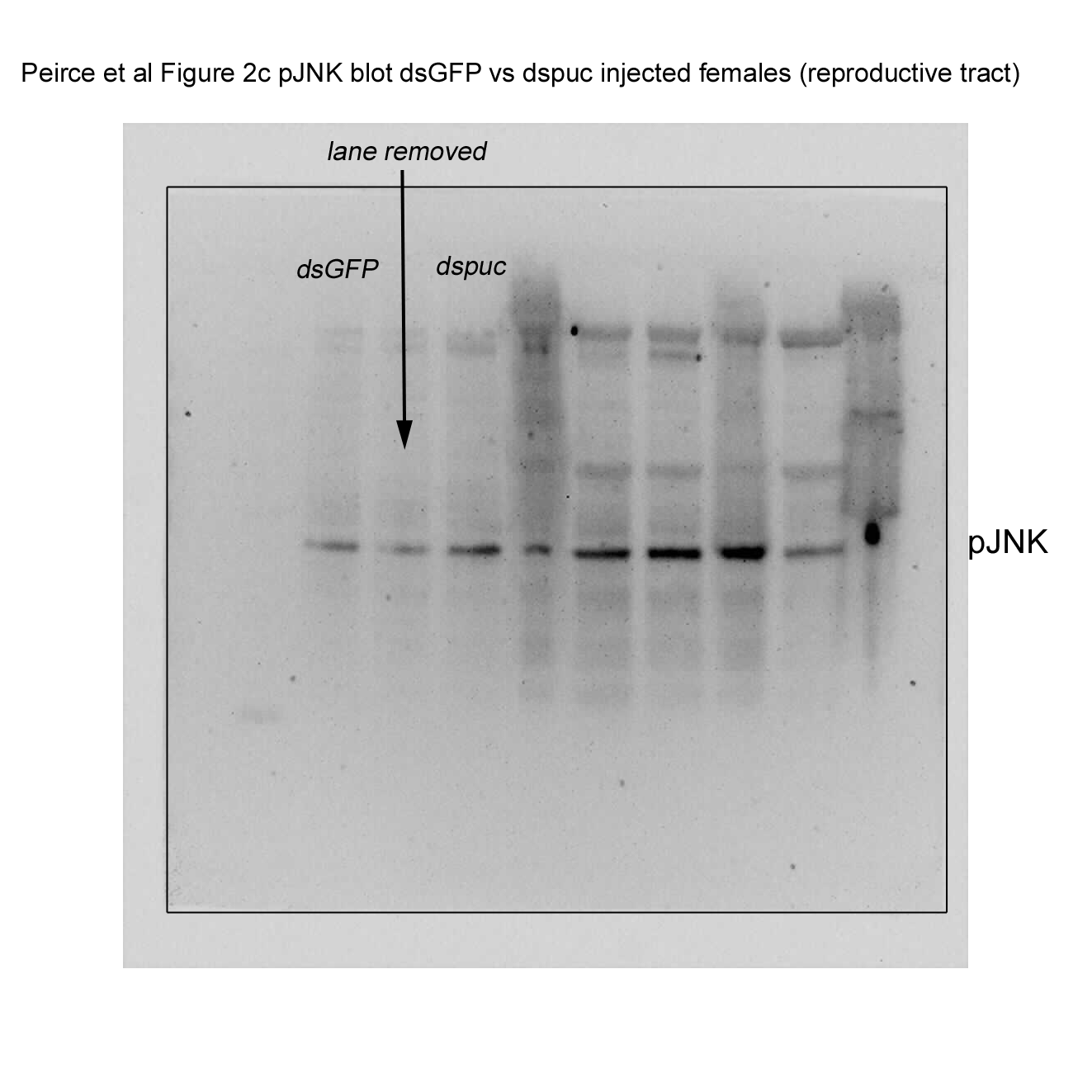


**Peirce et al Figure 2c**

actin re-probe (reproductive tract)


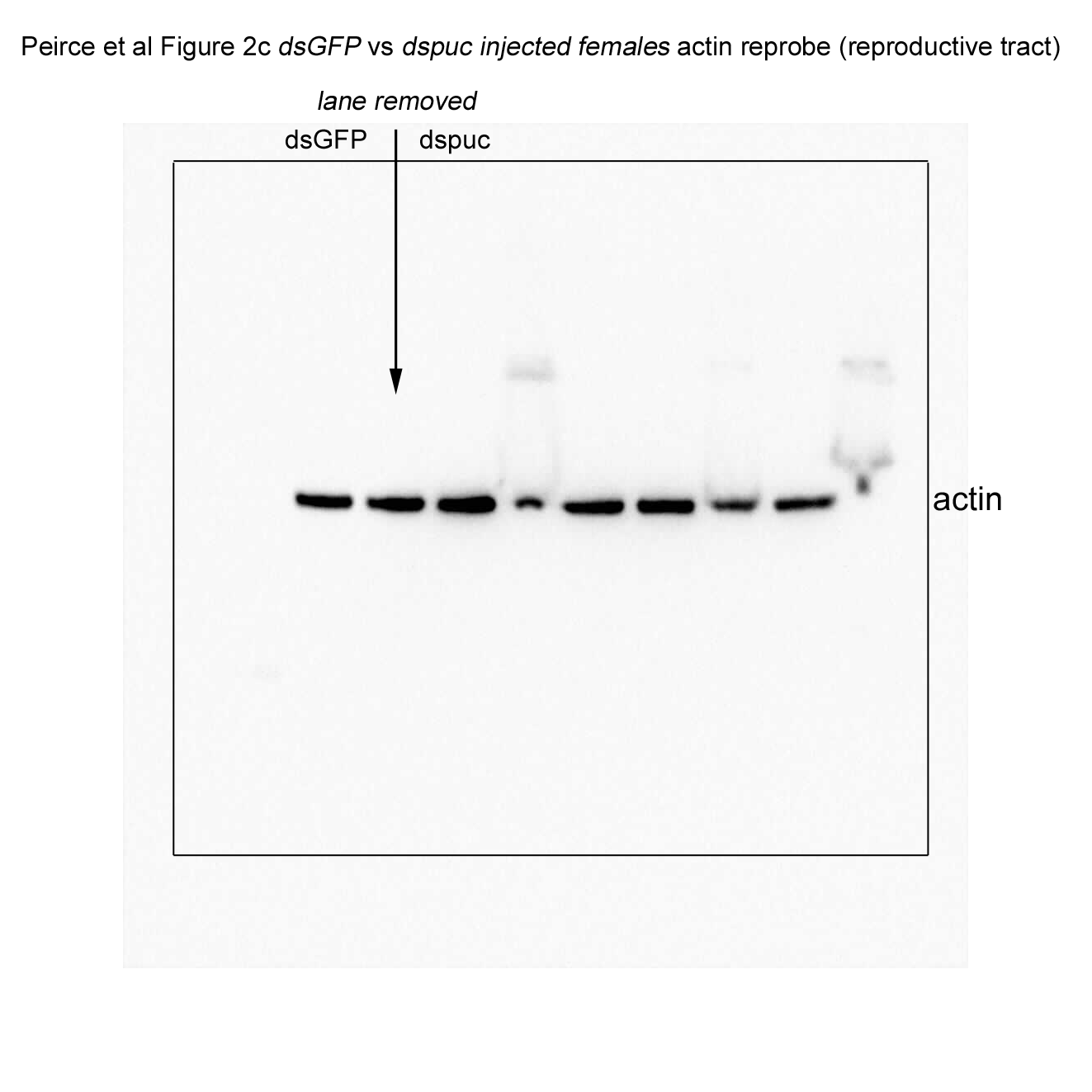


**Peirce et al Figure 2c**

Molecular weight ladder (Reproductive tract)


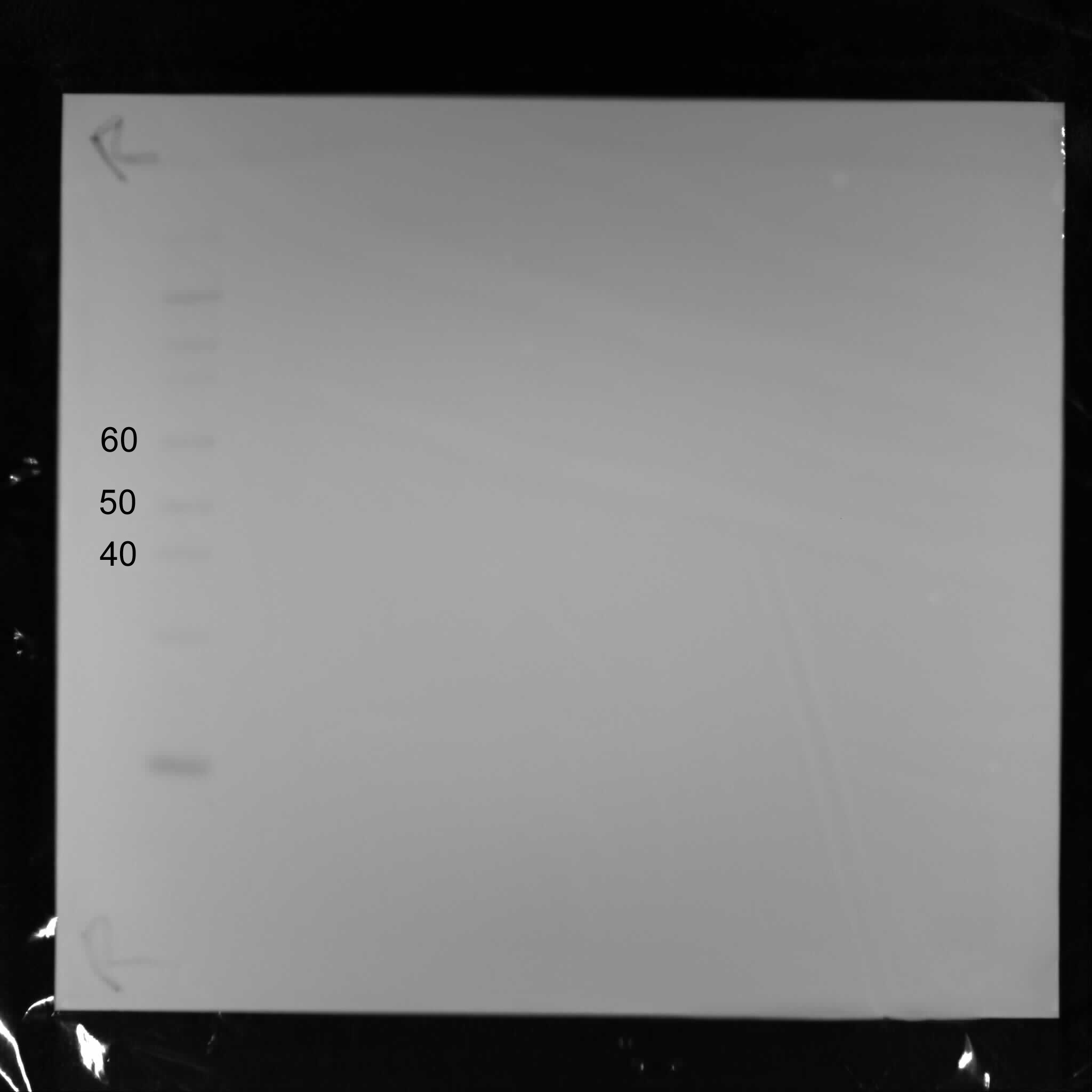


**Peirce et al Figure S1**

Three tissues, pJNK vs pERK

**
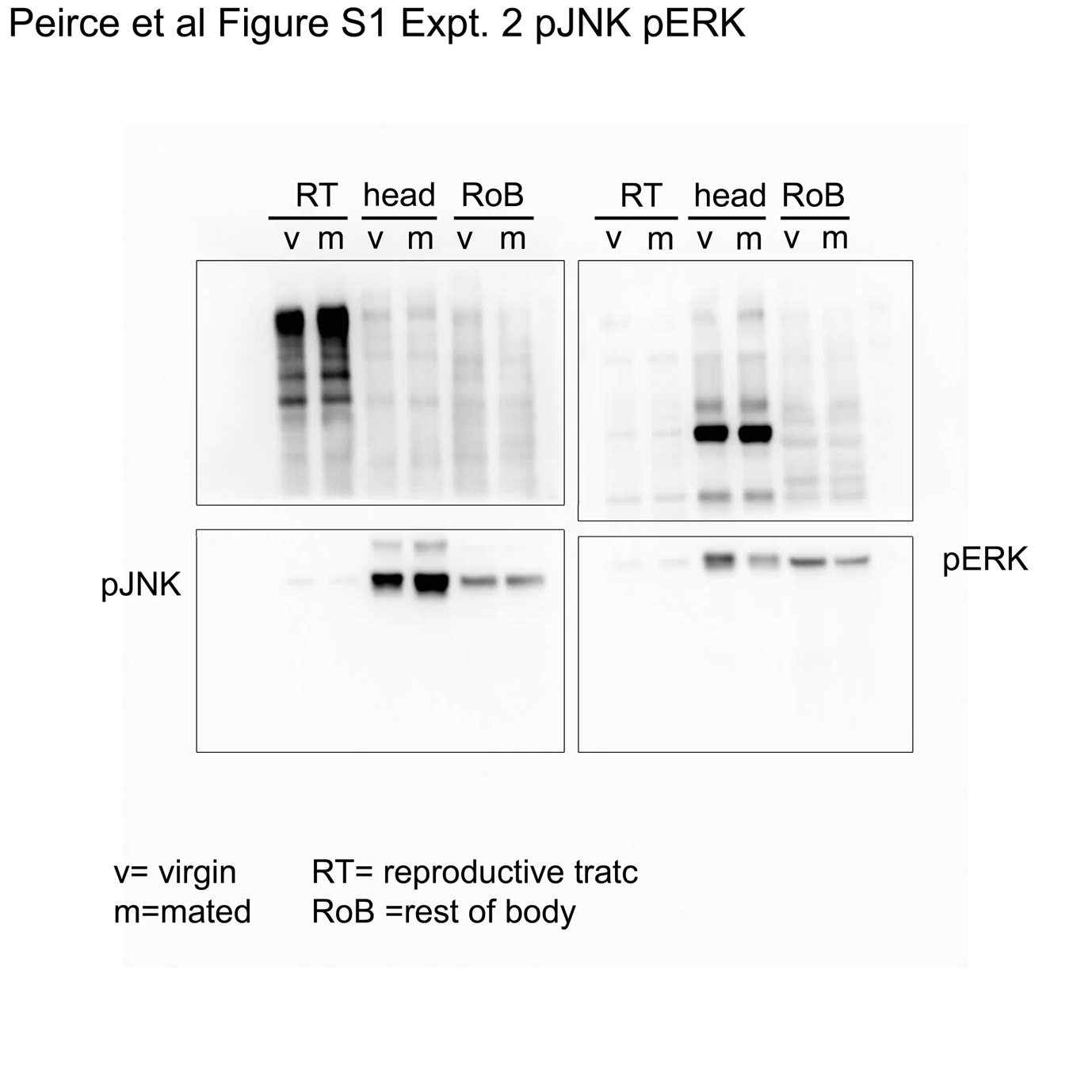
**

**Peirce et al Figure S1**

Three tissues, actin reprobe


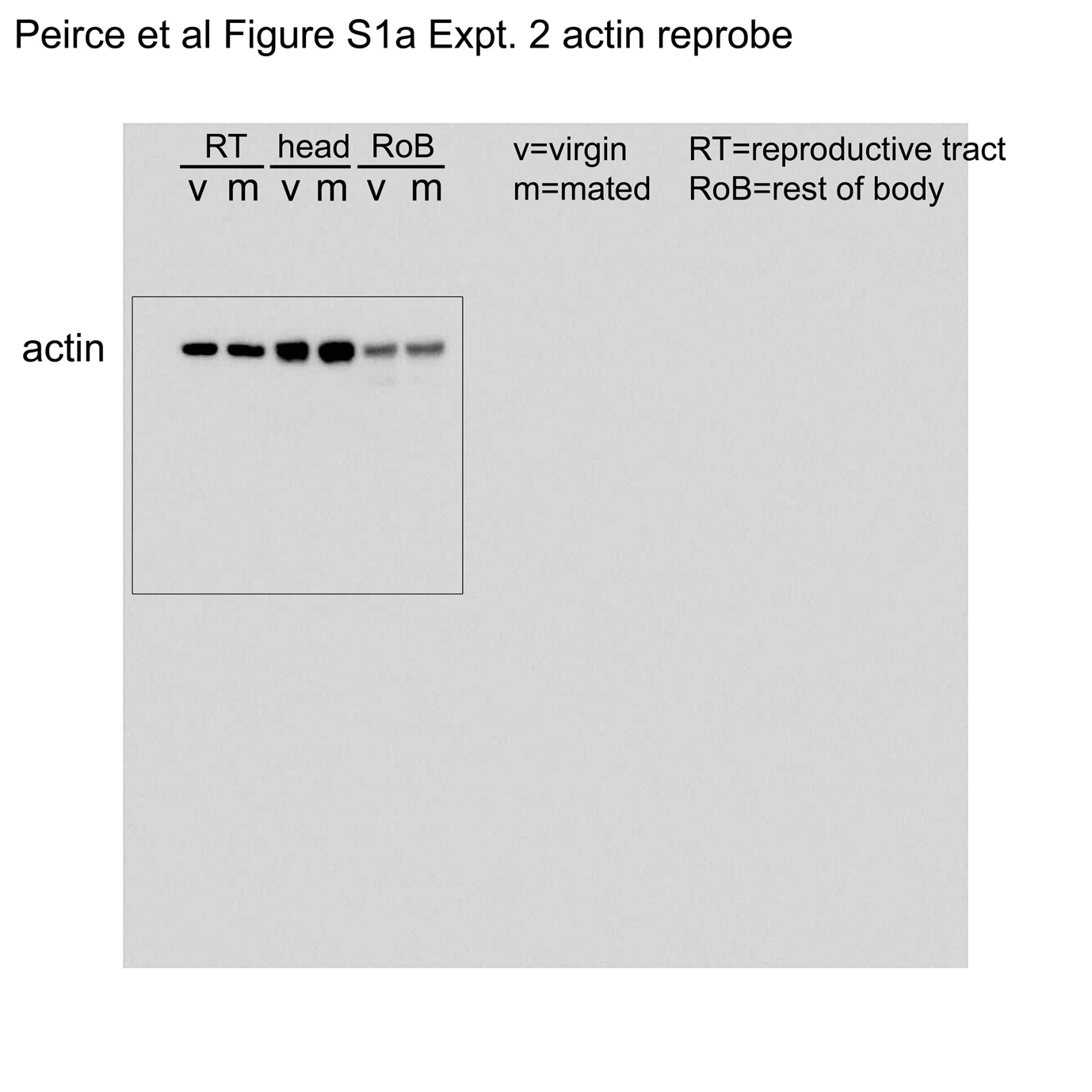


**Peirce et al Supplementary Figure 1**

Three tissues molecular weight ladder


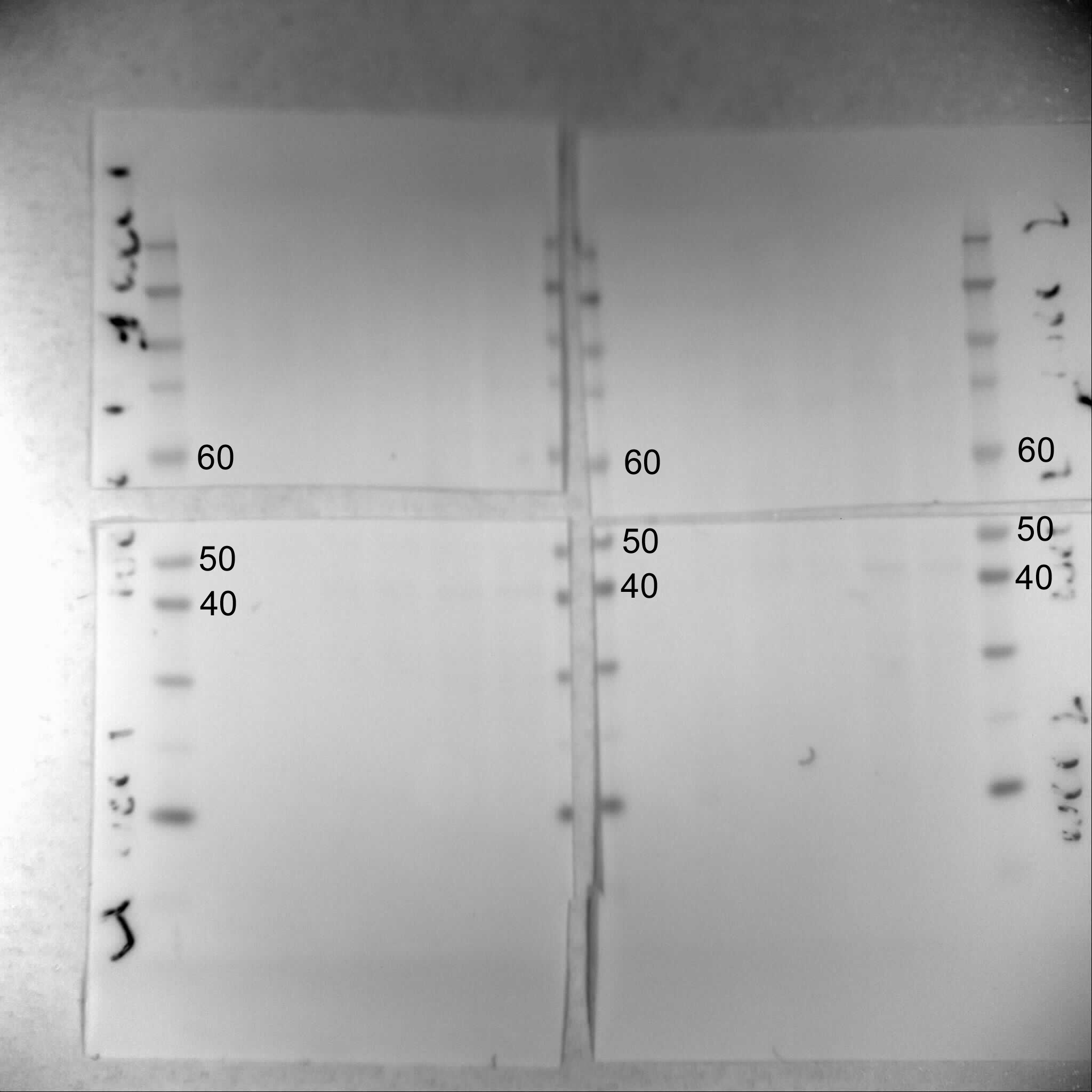


**Peirce et al Supplementary Figure 4**

pJNK (head)


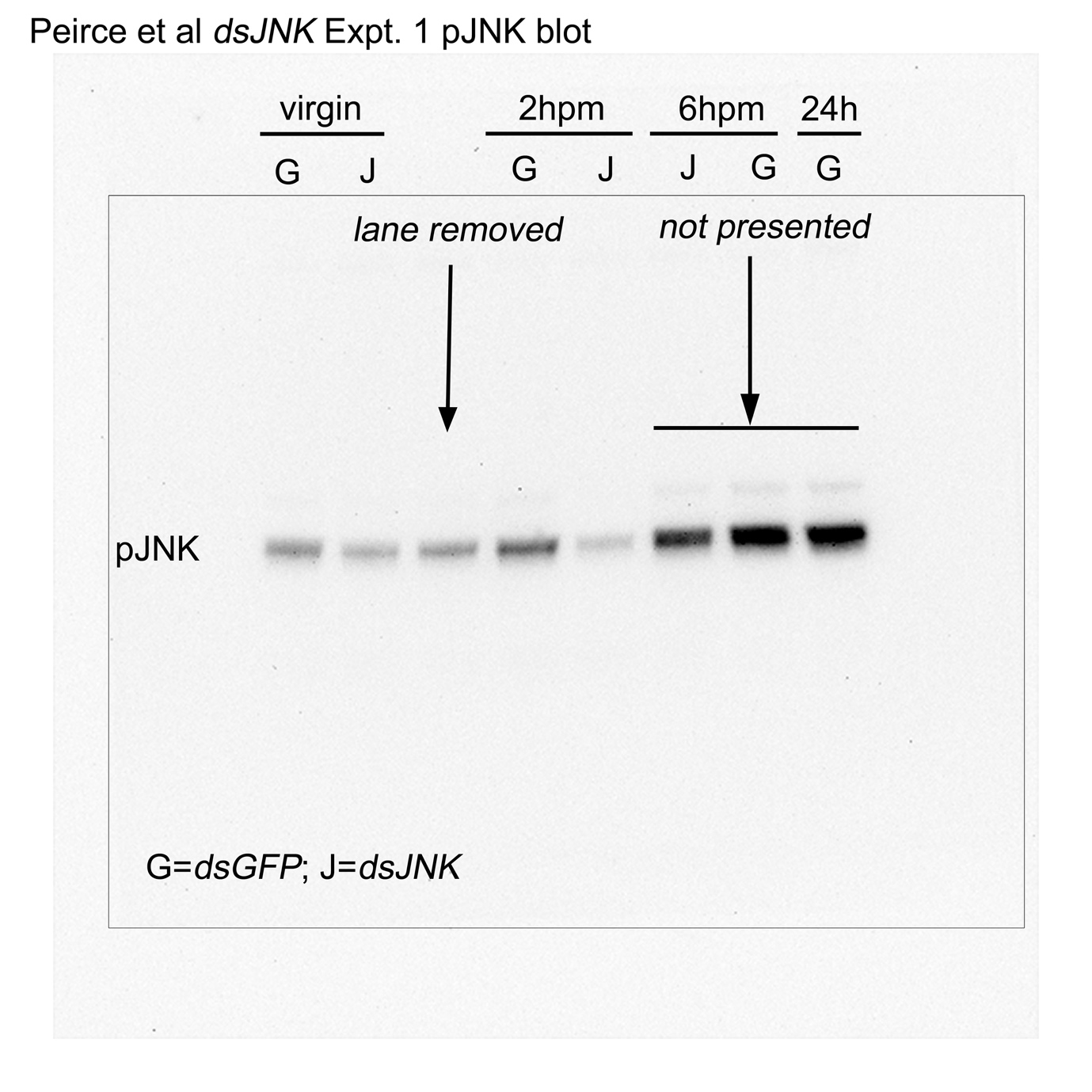


**Peirce et al Supplementary Figure 4**

Actin reprobe (head)


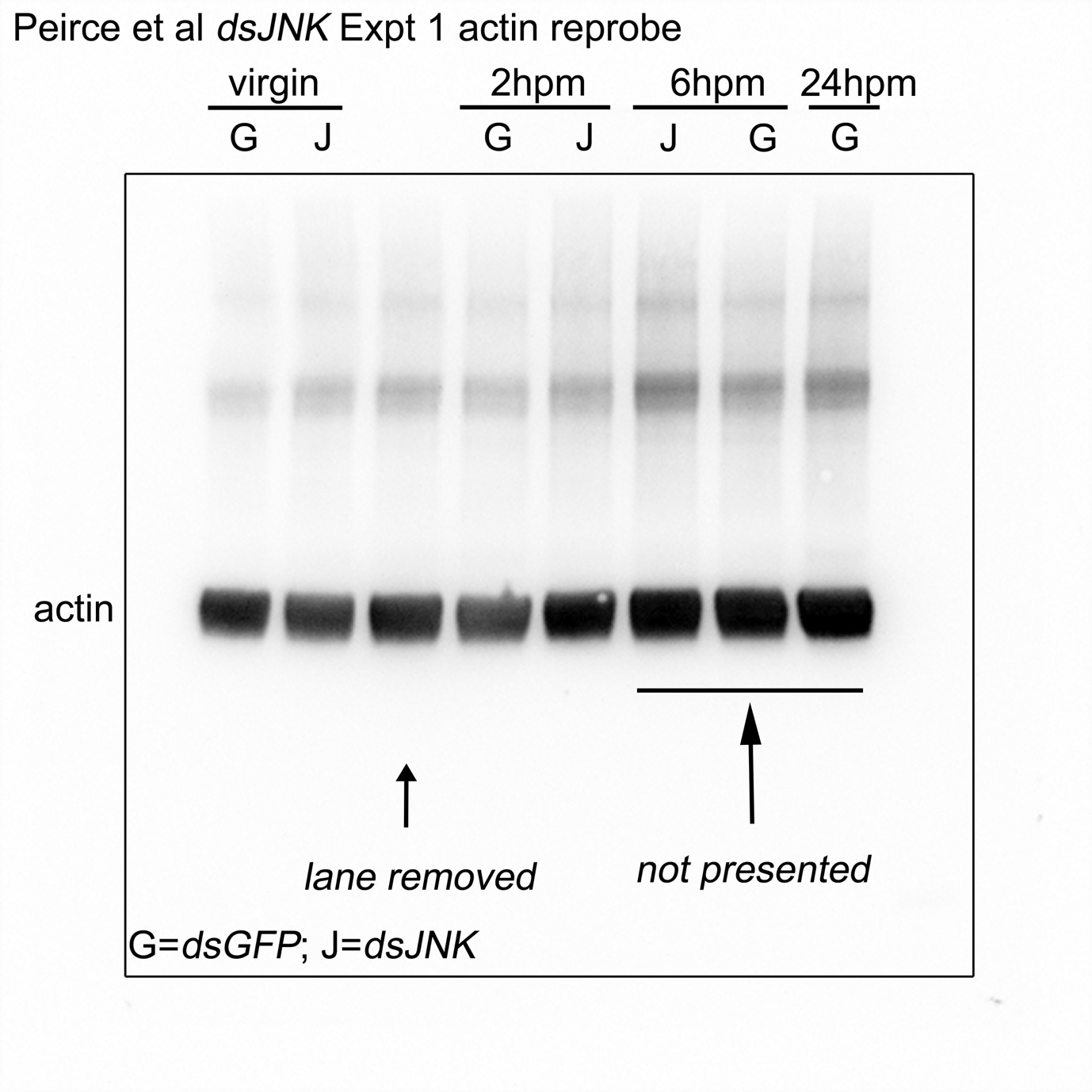


**Peirce et al Supplementary Figure 4**

Molecular weight ladder


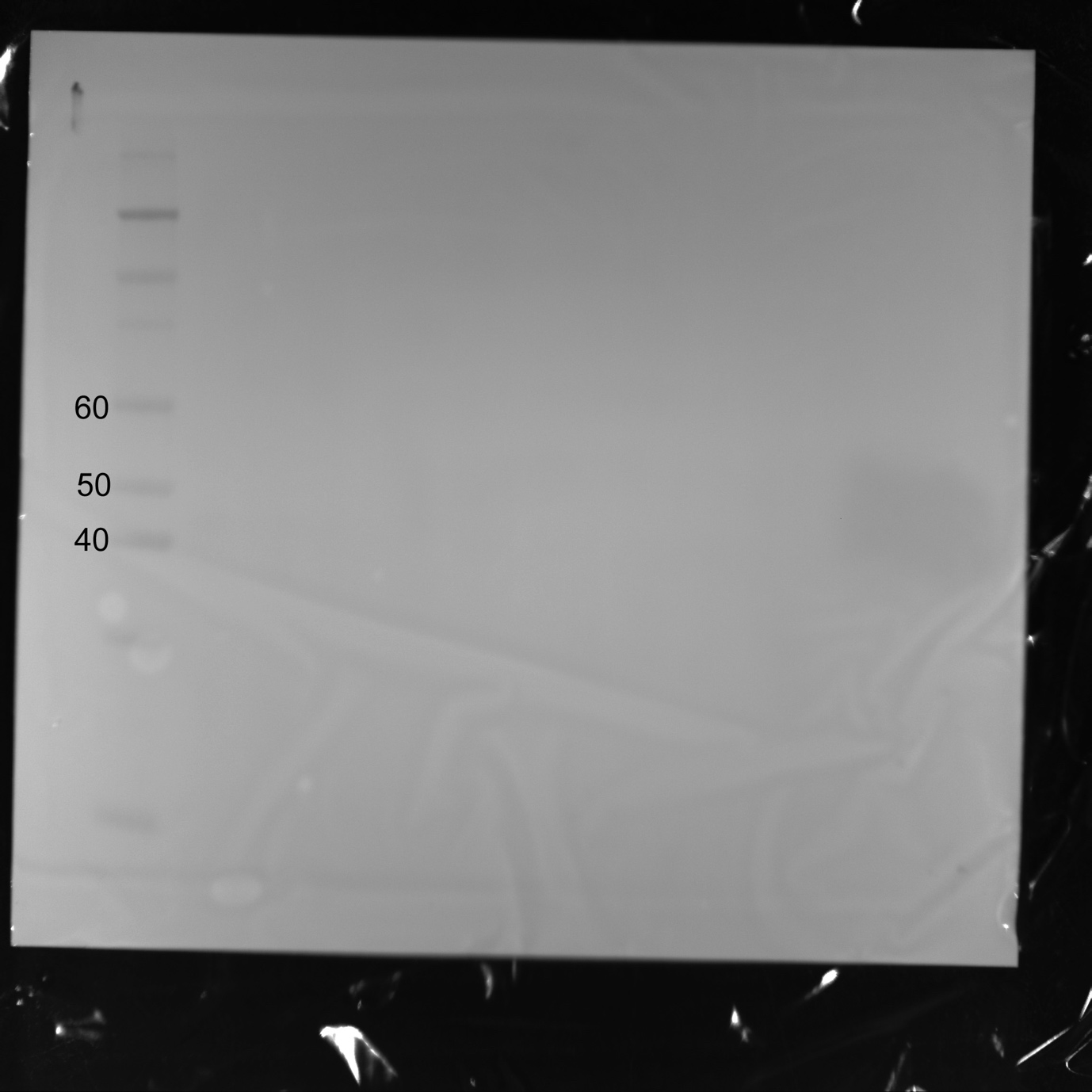

Supplement: Supplementary file 1 — Supplementary information [file 41598_2020_71291_MOESM1_ESM.docx]
